# Systematically Studying Kinase Inhibitor Induced Signaling Network Signatures by Integrating Both Therapeutic and Side Effects

**DOI:** 10.1371/journal.pone.0080832

**Published:** 2013-12-05

**Authors:** Hongwei Shao, Tao Peng, Zhiwei Ji, Jing Su, Xiaobo Zhou

**Affiliations:** 1 Department of Radiology, Wake Forest School of Medicine, Winston-Salem, North Carolina, United States of America; 2 Department of Modern Mechanics, University of Science and Technology of China, Hefei, Anhui, P.R. China; 3 Department of Translational Imaging, The Methodist Hospital Research Institute, Houston, Texas, United States of America; National Cancer Institute, United States of America

## Abstract

Substantial effort in recent years has been devoted to analyzing data based large-scale biological networks, which provide valuable insight into the topologies of complex biological networks but are rarely context specific and cannot be used to predict the responses of cell signaling proteins to specific ligands or compounds. In this work, we proposed a novel strategy to investigate kinase inhibitor induced pathway signatures by integrating multiplex data in Library of Integrated Network-based Cellular Signatures (LINCS), e.g. KINOMEscan data and cell proliferation/mitosis imaging data. Using this strategy, we first established a PC9 cell line specific pathway model to investigate the pathway signatures in PC9 cell line when perturbed by a small molecule kinase inhibitor GW843682. This specific pathway revealed the role of PI3K/AKT in modulating the cell proliferation process and the absence of two anti-proliferation links, which indicated a potential mechanism of abnormal expansion in PC9 cell number. Incorporating the pathway model for side effects on primary human hepatocytes, it was used to screen 27 kinase inhibitors in LINCS database and PF02341066, known as Crizotinib, was finally suggested with an optimal concentration 4.6 uM to suppress PC9 cancer cell expansion while avoiding severe damage to primary human hepatocytes. Drug combination analysis revealed that the synergistic effect region can be predicted straightforwardly based on a threshold which is an inherent property of each kinase inhibitor. Furthermore, this integration strategy can be easily extended to other specific cell lines to be a powerful tool for drug screen before clinical trials.

## Introduction

The basic components of biological systems, genes, proteins, metabolites and other molecules work together in a highly orchestrated manner within cells to promote normal development and sustain health [1]. Understanding how these interconnected components of biological pathways and networks are maintained in health, and how they become perturbed by genetic or environmental stressors and cause disease, is challenging but essential to developing new and better therapies to return perturbed networks to their normal state. To achieve this goal, the Library of Integrated Network-based Cellular Signatures (LINCS) project (http://lincs.hms.harvard.edu/) aims to develop a library of molecular signatures, based on gene expression and other cellular changes that describe the response of different types of cells when exposed to various perturbing agents, including siRNAs and small bioactive molecules. Diverse high-throughput screening approaches are applied in LINCS project to interrogate the cells, which provide molecular changes and intuitive patterns (gene or protein profile) of cell response for biologists. The data acquired from these approaches were collected in a standardized, integrated, and coordinated manner [2], [3] to promote consistency and comparison across different cell types. These data will also be made openly available as a community resource that can be easily scaled up and augmented to address a broad range of basic research questions and to facilitate the identification of biological targets for new disease therapies.

Nevertheless, it is not so straightforward for biologists to uncover the cell signaling and regulatory pathways from such abundance of information. As a result, it is increasingly recognized that mathematical approaches, such as statistical inference, graph analysis, and dynamic modeling, are desired to make sense of different observed patterns. In the past decades, substantial effort has been devoted to constructing and analyzing large-scale gene or protein networks based on different types of data and literature mining. Woolf et al. [4] used Bayesian approach to infer the signaling network responsible for embryonic stem cell fate responses to external cues based on measurements of 28 signaling protein phosphorylation states across 16 different factorial combinations of stimuli. The inferred network predicted novel influences between ERK phosphorylation and differentiation as well as between RAF phosphorylation and differentiated cell proliferation. The graph analysis, alone or combined with additional information regarding the network nodes, such as the functional annotation of the corresponding genes or proteins, provide testable biological predictions on several scales, from single interactions to functional modules. The functions of unannotated proteins can be inferred on the basis of the annotation of their interacting partners, as it was done for S. cerevisiae and Arabidopsis proteins using interaction, co-expression, and localization data [5]–[7]. A dynamic model that correctly captures experimentally observed normal behavior allows researchers to track the changes in the system’s behavior due to perturbations. Heinrich et al. [8] developed a mathematical theory that described the regulation of signaling pathways as a function of a limited number of key parameters. They found that phosphatases had a more pronounced effect than kinases on the rate and duration of signaling, whereas signal amplitude was controlled primarily by kinases. Morris et al. [9] proposed a novel approach, termed constrained fuzzy logic, to convert a prior knowledge network into a computable model. Then a context-specific network model can be created by training this model against the experiment data. These models shed light on the design principles of biological control systems but are rarely context specific and cannot be used to predict the responses of cell signaling proteins as well as phenotypes to specific ligands or compounds.

In cellular pathways, especially those involved in signal transduction, kinases are known to be the major regulators which can modify up to 30% of all human proteins. Deregulation of specific kinase activity has emerged as a major mechanism by which cancer cells evade normal physiological constraints on growth and survival. As a result, there are considerable efforts to develop selective small molecule inhibitors for a host of kinases that are implicated in different cancers [10]. In LINCS project, small molecule kinase inhibitors are also an area of focus in various perturbing agents tested. Thus, integration of different types of datasets in LINCS database will be a desired but challenge task to reveal the response of biological network perturbed by various kinase inhibitors. In this paper, we proposed a novel strategy to study the kinase inhibitor induced network signatures and assess the kinase inhibitor effect by considering both suppression effect on cancer cells and side effects on primary human hepatocytes *in silico*. This strategy integrated KINOMEscan data of kinase inhibitors, proliferation/mitosis imaging data of cancer cells and cue signaling response data of primary human hepatocytes in LINCS database to establish a systematical network model. To our knowledge, proliferation/mitosis imaging data was first used to establish the content-specific pathway model which can bridge the gap between specific kinase inhibitors and cell phenotypes. PC9 cell line was then chosen as an example of cancer cells for specific pathway development. Integrating this PC9 cell specific model with side effects on primary human hepatocytes, we can screen out the proper kinase inhibitors with optimal concentration levels to suppress the PC9 cancer cell proliferation while avoiding severe damage to primary human hepatocytes.

## Results

### Summary of the Proteomic Data Available in LINCS Database

**Figure 1:**
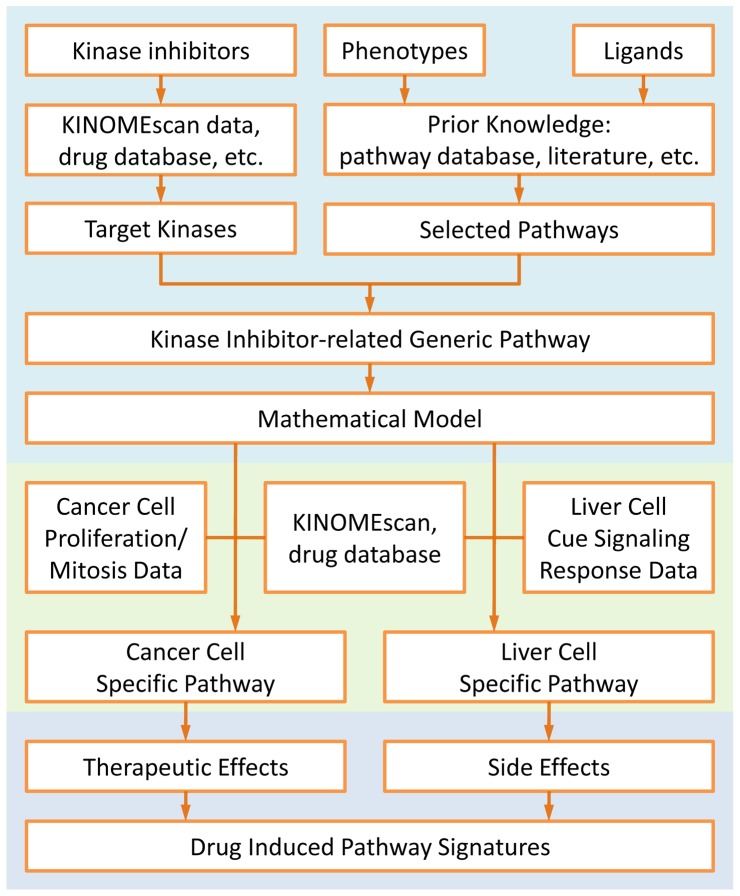
Flow chart of proposed system approach to investigate the kinase inhibitor induced pathway signature in both therapeutic and side effects.

Diverse high-throughput screen approaches were applied in LINCS project to investigate the cell response to various perturbations at different levels, such as gene expression, protein activity and cell phenotypes. Table 1 lists the datasets available in LINCS database except those at gene level. These datasets fall into three categories: a) KINOMEscan data [11] and KiNativ data [12] measure the interaction between inhibitors and target proteins; b) liver cell cue signal response data represent the protein phosphorylation states in the perturbed cells; c) other imaging data capture the response of cell phenotype to perturbation agents. In order to systematically reveal the kinase inhibitor induced intracellular network signature based on these datasets, we proposed a novel approach illustrated in Figure 1, which involved not only the suppressive effect on cancer cells but also the side effects on liver cells. Usually, cells can change their phenotypes through different pathways to respond the perturbation or stimulation from microenvironment. Thus, in our approach, we first determine the cell phenotypes, e.g. different phases in cell cycle, which are related to the kinase inhibitor we concern. Then several canonical pathways related to these phenotypes can be selected from literatures or pathway databases, such as IPA (Ingenuity® Systems, www.ingenuity.com) and KEGG [13], [14]. Meanwhile, proteins targeted by the kinase inhibitor can be listed according to the drug databases or KINOMEscan data. All the selected pathways are then filtered and those pathways which contain the proteins in this list will be integrated into a kinase inhibitor related generic pathway. Then, a mathematical model, which uses ordinary differential equations (ODEs) to represent the dynamics of the protein levels, can be developed based on this generic pathway. After trained by specific experiment data, the model can be used for capturing the kinase inhibitor induced signature of a content-specific pathway system. In this paper, cancer cell proliferation/mitosis data and liver cell cue signaling response data were used for training the mathematical model to generate the pathway for therapeutic effect and the pathway for side effects, respectively. Finally, overall considering these two pathways, the kinase inhibitor induced network signature can be studied from a holistic view. The detailed modeling procedure will be described in the following sections.

**Table 1:** Datasets in LINCS database.

Dataset	Description
KINOMEscan Data	A competition binding assay that is run for a compound of interest against each of a panel of 317 to 456 kinases.
KiNativ Data	A competition binding assay that is run for a compound of interest against each of a panel of 194 to 316 kinase labelling sites.
Apoptosis Imaging Data	Dose response of anti-mitotic compounds in human cancer cell lines at 24, 48, and 72 hours to determine their effects on apoptosis.
Growth Inhibition Data	A DNA stain based assay to determine cell viability following compound treatment.
Proliferation/Mitosis Imaging Data	Dose response of anti-mitotic compounds in human cancer cell lines at 24, 48 and 72 hours to determine effect on cell proliferation and mitosis.
Mitosis/Apoptosis Imaging Data	Dose response of anti-mitotic compounds in human cancer cell lines at 24, 48 and 72 hours to determine effect on apoptosis, mitosis and cell death.
Liver Cue Signal Response Data	Protein phosphorylation response of primary human hepatocytes and transformed liver cell lines to pairwise combinations of small molecule inhibitors and ligands.

### Binding Affinity of Kinase Inhibitor with its Targets

**Figure 2:**
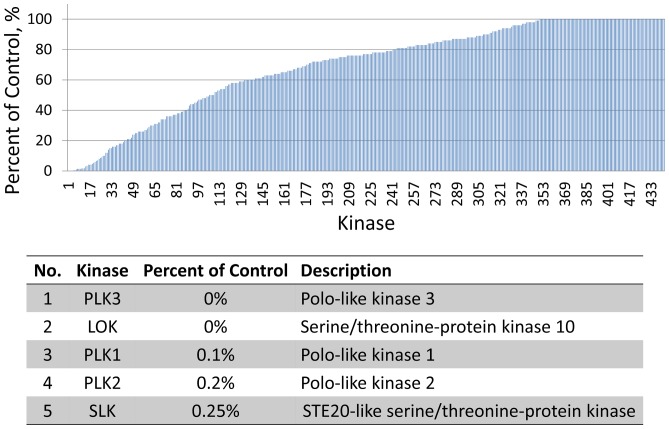
KINOMEscan data of GW843682 at 10 uM.

Most kinase inhibitors can interact with a wide range of proteins with different activities. As a result, determination of the binding affinity of a kinase inhibitor with different proteins will be the first step in the modeling system to connect the kinase inhibitor to cellular signaling pathway. A popular approach to test the binding affinity is KINOMEscan screen, which is based on a competition binding assay that is run for a compound of interest against each of a panel of over 400 kinases. The readout from assay is “percent of control”, where the control is DMSO and a 100% result means no inhibition of the kinase by the test compound. Figure 2 shows the KINOMEscan data of kinase inhibitor GW843682 at 10 uM in LINCS database. We can see that GW843682 at 10 uM concentration can bind with a large amount of proteins (∼80% of tested proteins), which demonstrates a complex perturbation on the signal transmission in the cell other than a single target modulation. The table in Figure 2 lists six kinases which have the highest binding affinity with GW843682. As the primary targets of GW843682, PLK family kinases indeed show strong inhibition by GW843682. The other two kinases, LOK and SLK, are also PLK related proteins which function by association with and phosphorylating PLK family kinases [15], [16]. Since PLK family kinases are the early triggers for phase transition in cell cycle, cell cycle arrest could be induced with the existence of GW843682.

**Figure 3:**
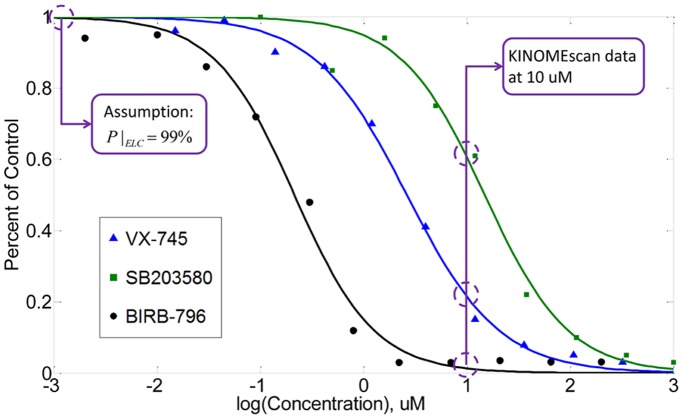
Binding affinity curves of p38α bound by three kinase inhibitors at different concentrations.

**Figure 4:**
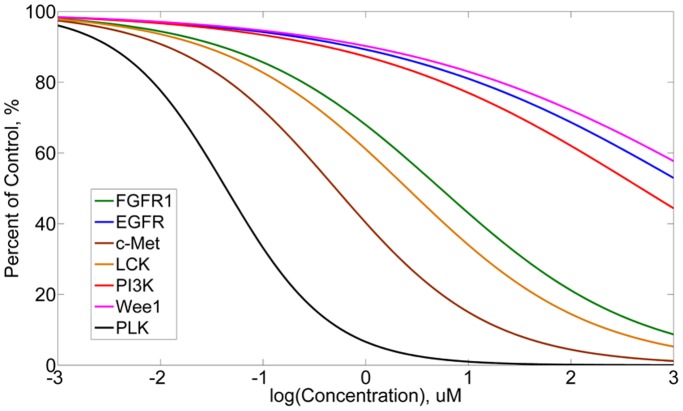
Binding affinity prediction for GW843682 with seven target proteins.

The binding affinity of kinase inhibitors was only tested at one concentration in LINCS KINOMEscan dataset. Thus, extension of the kinase inhibitor binding affinity at different concentrations becomes necessary for prediction of the cell responses to kinase inhibitors with specific dosages. Fortunately, binding affinity curves usually show a similar shape for different kinase inhibitors if they can bind with a given protein. Figure 3 shows the binding affinity data of three kinase inhibitors with their primary target p38α [11]. It can be seen that a sigmoid curve can properly fit the experimental data. Thus, for each protein which can be bound by the inhibitor, a sigmoid function is adopted in this paper to represent the relationship between “percent of control” and the kinase inhibitor concentration. The parameters in this function are acquired based on the KINOMEscan data at 10 uM and an acceptable assumption that extremely low concentration (ELC) of kinase inhibitor results in a high “percent of control” approaching 100% (details are described in materials and methods). Figure 4 shows the predicted curves for those proteins which can be bound by GW843682. In this prediction, the ELC in our assumption was set to be 0.00001 uM, which was much lower than the minimum concentration 0.00019 uM used in cancer cell proliferation/mitosis imaging assay. As expected, since PLK is the primary target of GW843682, its capability of activating the downstream proteins decreases rapidly while the concentration of GW843682 increases. It is also indicated that 10 uM GW843682 is enough to completely suppress signaling transduction through PLK. For other target kinases, extremely high concentrations are required to strongly suppress their functions. We can see that even the concentration of GW843682 achieves 1000 uM, the percentage of unbound Wee1 is still larger than 50%. These binding affinity curves can provide the connection between the kinase inhibitors and signal transmission in the pathway network.

### Cell Cycle Related Pathway for Cancer Cells

**Figure 5:**
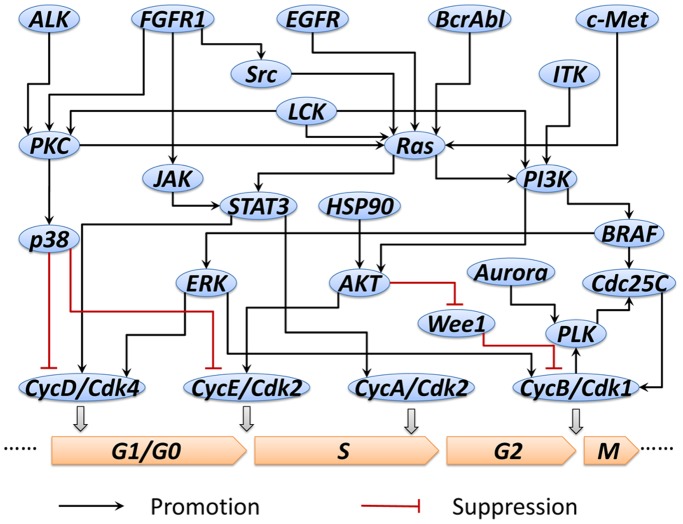
Cell cycle related pathway extracted from literatures and pathway databases.

Abnormal cell expansion is a prominent hallmark in cancer development. Targeting the cell cycle related pathway is a potential way for kinase inhibitors to decelerate or suppress the unregulated cancer cell growth. In LINCS project, dose responses of eight kinase inhibitors in seventeen human cancer cell lines were tested to determine their effect on cell proliferation and mitosis. Cellular Imaging technique was applied at different time points to capture the cell response and five readouts were acquired from these images as listed in Table 2 (details are described in materials and methods). All these readouts are referring to the number of the cells in different cell cycle stages. Thus, we selected the cell cycle related pathways from literatures and pathway databases, such as IPA and KEGG. Then those pathways, which contained the primary targets of different inhibitors in LINCS KINOMEscan data, were screened out and integrated into a kinase inhibitor related pathway, as shown in Figure 5. In this pathway map, 26 proteins and complexes were involved. Perturbations by kinase inhibitors on these proteins or complexes will be transmitted through protein-protein interactions and then regulate the production of cyclin-dependent kinase complexes, e.g. CycE/Cdk2. These complexes are necessary in sustaining the normal process of cell cycle and lack of these complexes will result in cell cycle arrest. Since few cells stay in the quiescent phase called G0 for the cell lines in growth media, these cells are not considered separately and incorporated into G1 phase in this work. As a result, the final output of this pathway system will be the cell number in four distinct phases of cell cycle, G1, S, G2 and M. Based on this pathway network, a mathematical model was developed using hill function as described in materials and methods.

**Table 2:** Readouts from proliferation/mitotic imaging data.

Readout	Description
Cell Count	The total number of cells (nuclei) stained with Hoechst 33342 and detected in the DAPI channel
EdU positive cells	The percentage of actively proliferating cells that have average FITC (EdU) intensity above the EdU threshold
Mitotic cells	The percentage of cells that are MPM2-positive
Non-mitotic cells that are EdU-positive	The percentage of EdU-positive cells within the non-mitotic population
Mitotic cells that are EdU-positive	The percentage of EdU-positive cells within the mitotic population

### PC9 Cell Line Specific Pathway Network

**Figure 6:**
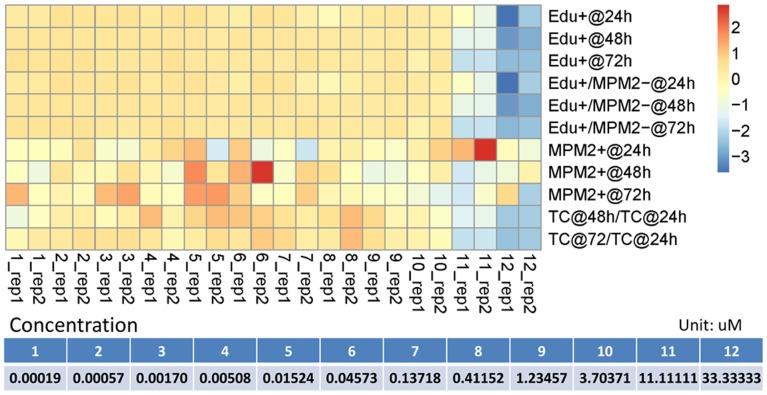
Heatmap for proliferation/mitosis imaging data of PC9 cell line exposed to GW843682.

**Figure 7:**
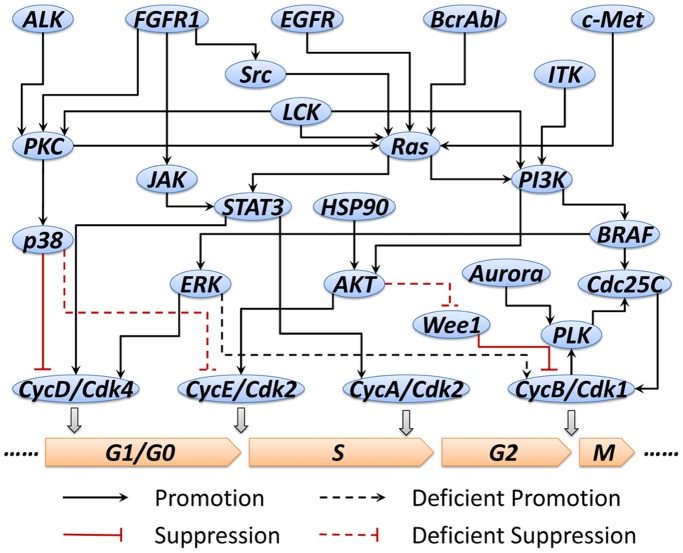
PC9 cell line specific pathway.

In order to obtain the content-specific pathway model, the proliferation/mitosis imaging data of a non-small cell lung cancer (NSCLC) cell line, PC9, exposed to kinase inhibitor GW843682 with twelve concentrations was used (Figure 6, data for each readout have been scaled for visualization). In this data, we can see that remarkable effect of the GW843682 on PC9 cell line emerged when the concentration level achieved 11.11111 uM. Using the predicted values of “percent of control” for 12 different concentration levels tested in the PC9 proliferation/mitosis imaging assay, we trained the cell cycle related pathway model to fit these readouts from the assay based on a two stage approach described in materials and methods. After the first stage of the parameter estimate approach, we can obtain a specific pathway network for PC9 cell line, as shown in Figure 7. Compared to the original pathway in Figure 5, we can see that the links between AKT and Wee1, p38 and CycE/Cdk2, as well as ERK and CycB/Cdk1 are deficient, which means that these signaling cascades are not as significant as other interactions in modulating the proliferation and mitotic progression of PC9 cell line. In these deficient links, AKT-dependent phosphorylation at Ser642 promotes a change in Wee1 localization from nuclear to cytoplasmic and this relocation will make cells enter G2/M checkpoint before mitosis [17]. Another link between p38 and CycE/Cdk2 is also involved in G1/S checkpoint [18]. Loss or attenuation of these cell cycle checkpoint functions can compromise the fidelity of DNA which has already been identified as a risk of developing lung cancers [19]. In other words, the deletion of these links will attenuate the cells’ capability of controlling the abnormal expansion of cell number. The resistance of signaling transmission in these links is a signature of PC9 signaling pathway and it reveals a potential mechanism how PC9 cancer cells altered its signaling pathway for rapid expansion of themselves.

**Figure 8:**
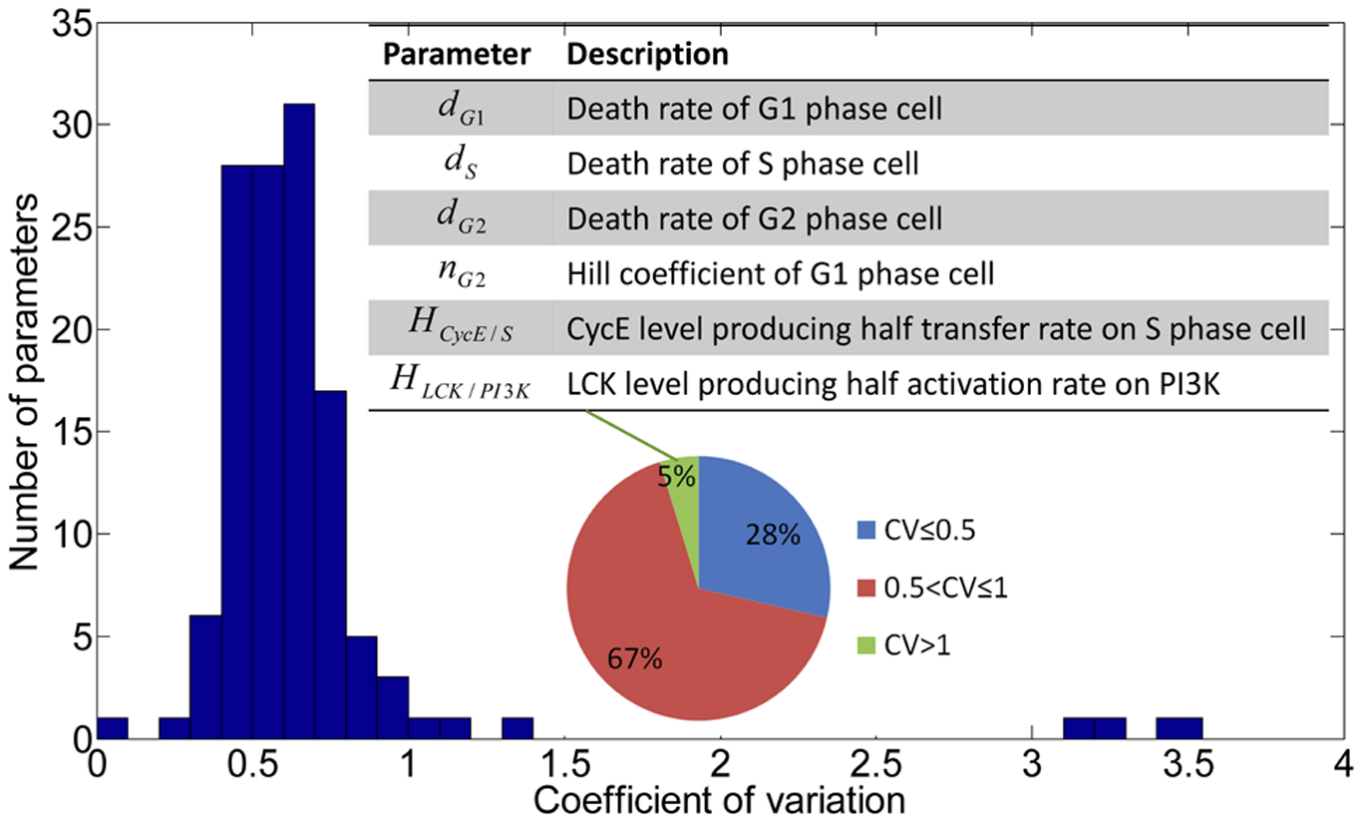
Coefficient of variation analysis for parameters in PC9 cell line specific pathway.

Based on this specific signaling pathway, we refined those non-zero parameters in the second stage. However, estimated parameters still varied in these repetitions. This might be caused by multimodal of the objective function or non-determinacy of the parameters in the mathematical model based on current dataset. As a result, coefficient of variation (CV) is used to investigate which parameters are determinable in this model. CV is a normalized measure of dispersion of a probability distribution of a variable, which is defined as the ratio of the standard deviation to the mean. Figure 8 shows the CV distribution of nonzero parameters in the mathematical model. It is indicated that most of the parameters (∼95%) can be considered to be identifiable since their CV values are not greater than 1 [20]. The rest parameters include 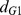, 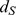, 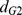, 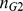, 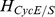 and 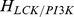. In these parameters, we found that the mean values of 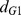, 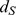, 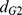 and 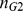 are very small, but not small enough to be considered as zero in the first stage of the estimate approach. This is a factor that can result in such kind of larger CV. As the variation of parameters shown in this system, such sloppy parameter estimates are in fact a consistent feature of systems biology models [21], and may reflect underlying robustness of biological networks, which always have a tolerance against a significant fraction of perturbations in cellular microenvironment [22].

### Prediction Capability of PC9 Cell Specific Pathway Model

**Figure 9:**
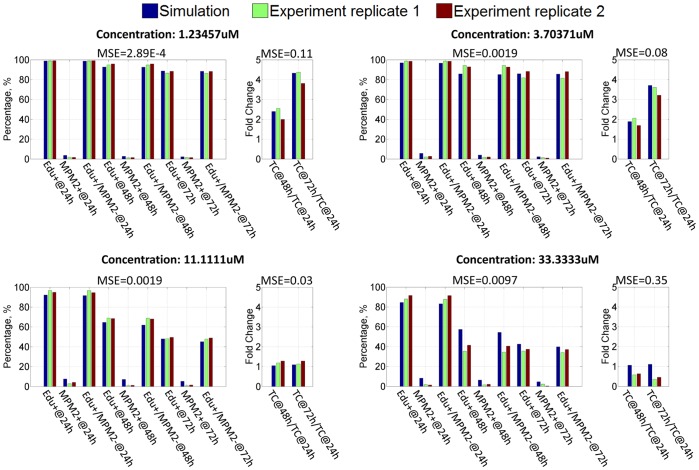
Comparison between simulation results and proliferation/mitosis data of PC-9 cell line. MSE: Mean square error.

**Figure 10:**
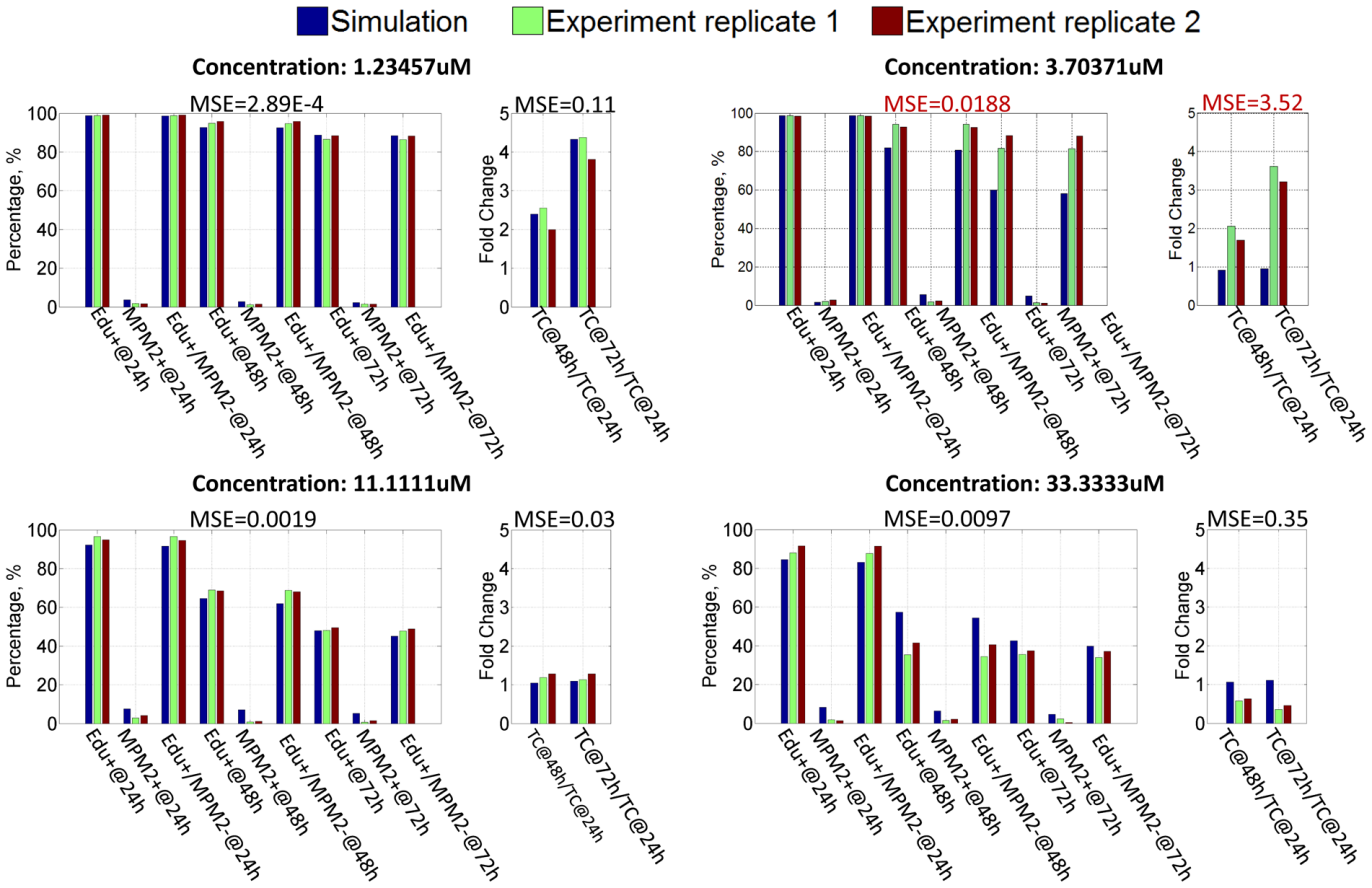
Leave-one-out cross-validation for PC9 cell line specific pathway model.

After the second stage of the parameter estimate, the parameter set which can produce the minimum error between the simulation results and experimental observations was selected as the final estimate. Figure 9 shows the simulation results when PC9 cells are treated by GW843682 with four concentration levels: 1.23 uM, 3.70 uM, 11.11 uM and 33.33 uM (Results for other concentration levels are shown in Figure S1). The simulation results represent a proper fitting to the experimental data with low mean square errors. In order to verify the prediction ability of this pathway model, leave-one-out cross-validation was employed. In each repetition, the observation in each condition is used once as the validation data while using the remaining observations to train the model. Figure 10 shows the predicted profiles for four conditions which are left out in turn for validation (Predicted profiles for other conditions are the same as the profiles in Figure S1). It demonstrates that in most conditions, a reliable prediction can be achieved to represent the validation data based on the pathway model trained by the remaining data. Nevertheless, for the condition with 3.70 uM concentration level, mean square error between the prediction and validation data is a little bit larger than those in other conditions. This bias is due to the sparse sampling in the region around 3.70 uM where PC9 cell response is very sensitive to the concentration level of GW843682.

**Figure 11:**
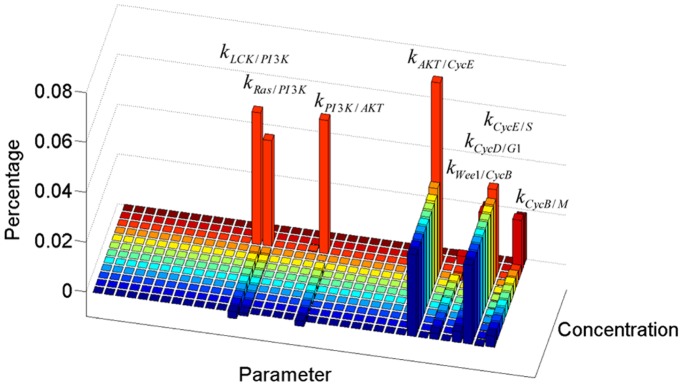
Sensitivity analysis of parameters in PC9 pathway model. Each parameter is increased by 2% of its estimated value.

Figure 11 shows the sensitivity analysis of the parameters representing activating rate or inhibiting rate at different concentration levels (Sensitivity analysis for other parameters is shown in Figure S2). This sensitive analysis demonstrates that the system model developed for the PC9 cell line pathway is rather robust for most parameters, which is consistent with the CV analysis. The sensitive parameters include 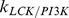, 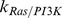, 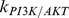, 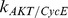, 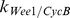, 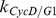, 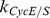 and 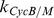. In these parameters, 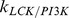, 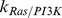, 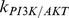 and 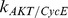 are all involved in the PI3K/AKT pathway, which has been confirmed to be associated with survival, proliferation, and invasiveness of most tumour cells [23], [24]. Especially under high concentration of GW843682, we can see that the alteration in PI3K/AKT pathway will modulate the proliferation process more sensitively. As a result, suppression of PI3K/AKT pathway activity can be considered as an assistant approach to achieve a more effective therapeutic result while using GW843682 treatment. Other sensitive parameters are all the downstream proteins close to cell cycle process, which reveals a higher tolerance to perturbation in upstream network than downstream network.

### Primary Human Hepatocyte Specific Pathway

The liver is the principal organ that is capable of converting drugs into forms that can be readily eliminated from the body. However, the liver is also susceptible to the toxicity of these accumulated drugs, known as hepatotoxicity [25]. Even when the drug is introduced within therapeutic ranges, it may also cause the damages to liver functions. A huge number of drugs have been implicated in causing liver injury [26] and it is the most common reason for a drug to be withdrawn from the market. As a result, assessment of their side effects on the liver cells after the treatment will provide useful information for drug prescreen before clinical trials. In this paper, we also established a liver cell specific signaling pathway by integration of multiplex data (KINOMEscan and cue signal response data) to investigate the kinase inhibitor induced side effects on normal function related proteins in liver cells.

**Figure 12:**
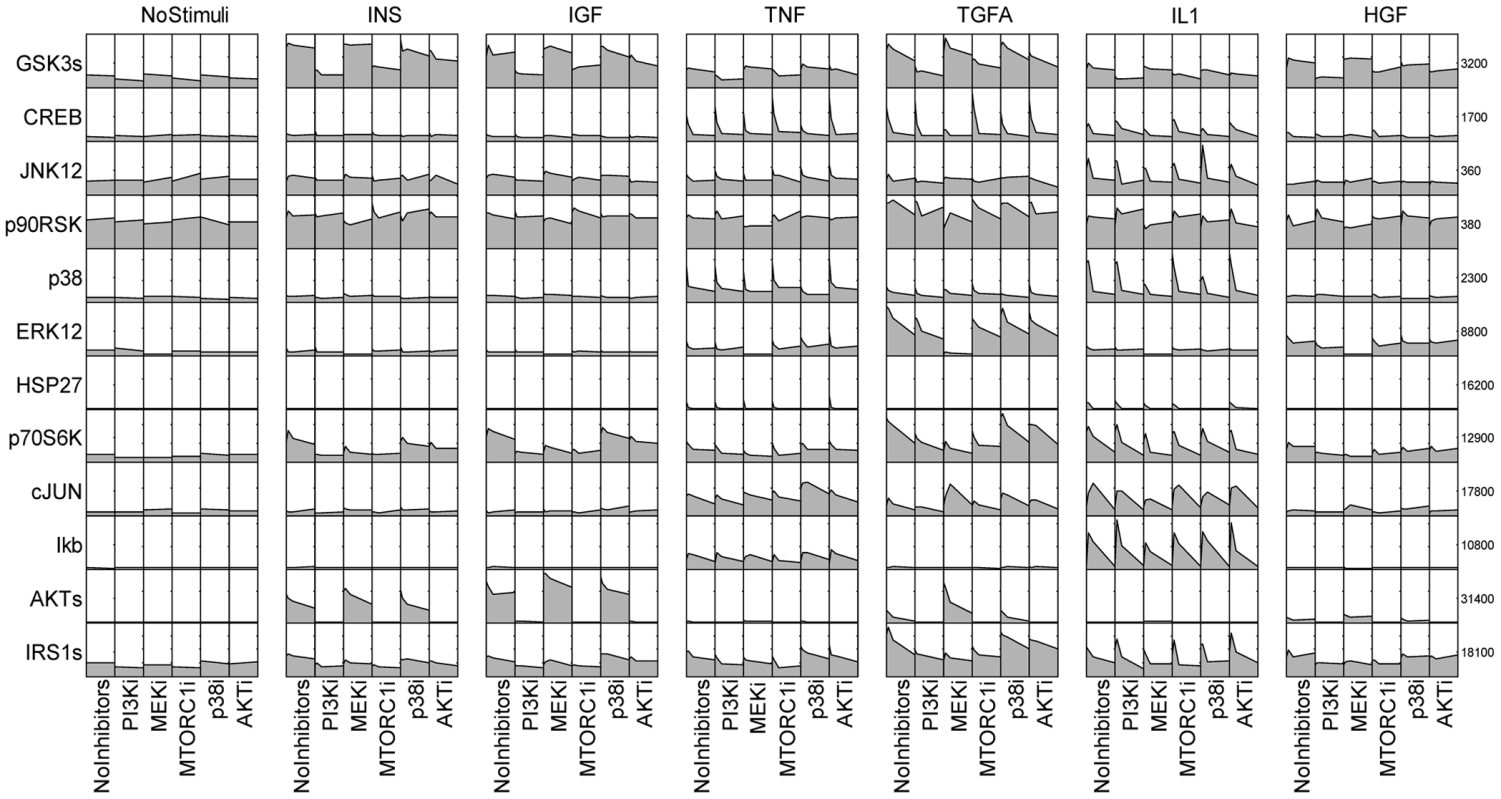
Cue signal response data of primary human hepatocyte. Each column represents a treatment condition which combines an inhibitor (or no inhibitor) and a ligand (or no ligand) while each row represents the measurement on each protein. The curve in each box shows the protein level variation at four time points.

Construction of the liver cell specific signaling pathway was based on the cue signal response data of primary human hepatocytes in LINCS database. In this assay, cells were explored to the pairwise combinations of 5 small molecule inhibitors and 6 ligands. Then phosphorylation states of 12 intracellular proteins were monitored by bead-based ELISA assay at four time points which were 10 min, 30 min, 90 min and 360 min. Figure 12 shows this dataset visualized by DataRail software [2]. Each column represents a treatment condition which combines an inhibitor (or no inhibitor) and a ligand (or no ligand) while each row represents the measurement on each protein. The curve in each box shows the protein level variation at four time points.

**Figure 13:**
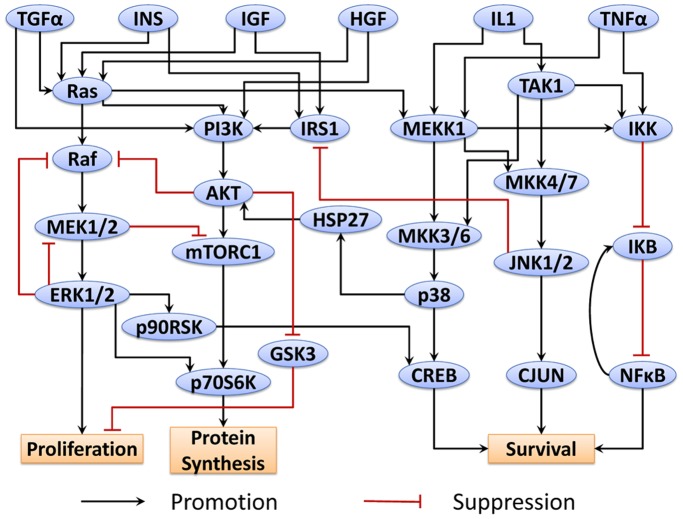
Cue signal response data-related pathway for primary human hepatocytes.

**Figure 14:**
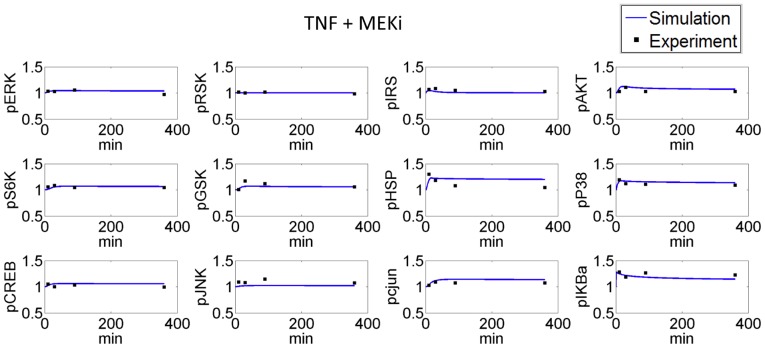
Comparison between simulation results and Cue signal response data of primary human hepatocyte exposed to a combination of TNF and MEK inhibitor.

According to the proteins monitored in the assay, a series of related canonical pathways was filtered based on the prior knowledge from literatures and pathway databases. Then we simplified the pathway network to contain only measured and perturbed proteins as well as any other proteins necessary to preserve logical consistency between those that were measured or perturbed, resulting in a compressed pathway shown in Figure 13. This pathway contains several well-known pathways as well as their crosstalks, such as PI3K/AKT pathway and p38 MAPK pathway. Inhibitor target proteins in these assays are PI3K, MEK, MTORC, p38 and AKT. Perturbations on these proteins will modulate the downstream transcription factors which will finally affect several normal functions, such as protein synthesis and cell survival. Based on this generic pathway, mass action law was used to develop the mathematical model. Primary human hepatocyte specific pathway model was then established by minimizing the difference between protein levels from mathematical model and ELISA data using the approach described in Materials and Methods. Figure 14 shows the simulation results when primary human hepatocytes are exposed to MEK inhibitor and TNF (Simulation results in other conditions are shown in Figure S3). Due to the variation between readouts from a large number of conditions and the simplified pathway model, several predications in these figures do not match the experimental dataset very well, such as AKT level in different conditions. However, this pathway model still captures the dynamic features of most protein levels in most conditions and can be used to predict the response of primary human hepatocytes to various perturbations.

### Effect Index Provides Combination of Therapeutic Effect and Side Effect

**Figure 15:**
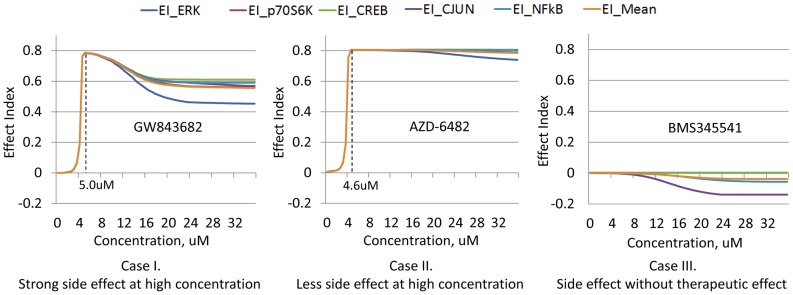
Effect indexes of three typical kinase inhibitors. I) Strong side effect at high concentration. II) Less side effect at high concentration. III) Side effect without therapeutic effect.

In order to comprehensively assess the effect of kinase inhibitors in treating the cancer disease, it is necessary to combine both the therapeutic effect and side effect at system level. As a result, we defined an evaluation criterion for the kinase inhibitors using an effect index which is a ratio between therapeutic effect and side effect. Figure 15 shows the five normalized effect indexes and an averaged effect index of three different kinase inhibitors with different concentrations on PC9 cell line, which can represent three typical cases of kinase inhibitor induced effect. For GW843682, when the concentration is extremely low, there is almost no effect on both cancer cells and liver cells. However, once the concentration achieves 2.5 uM, the integrated effect increases rapidly and reaches the peak value at 5 uM. This is due to the strong suppression on the cancer cell proliferation with slight damage to normal function of liver cell. If the concentration continues to increase, side effect of the kinase inhibitor emerges and plays a comparable role with the therapeutic effect. Thus, the integrated effect index begins to decrease. Finally, the integrated effect index will decrease to a constant value when the concentration reaches an extremely high level, which can result in the maximum suppression on both cancer cells and liver cells. In a word, along with the incensement of concentration, side effect will come up with a delay to the therapeutic effect. In this case, an optimal concentration can be chosen as the concentration producing the peak value of integrated effect index, which is 5 uM for GW843682. For AZD-6482, it shows similar curve with GW843682 before achieving the peak value. However, when the concentration continues to increase, the effect index will stay at the peak level with slight decrease, which means that the kinase inhibitor causes tiny side effect on liver cells while compared with the suppressive effect on PC9 cancer cells. As a result, it is reasonable to choose the minimum concentration producing the peak value of integrated effect index as the optimal concentration for this case, e.g. 4.6 uM for AZD-6482. In the last case, we can see that with the increase of BMS345541 concentration, the effect indexes stay at zero or even become negative. This indicates that the kinase inhibitor has less suppressive effect on this cancer cell line while only exhibits side effect on liver cells. Obviously, since this kinase inhibitor cannot be used to suppress the cell expansion of this cancer, no optimal concentration will be suggested. However, for the consistency with previous cases, we defined 0 uM as the optimal concentration for this case in the paper.

**Figure 16:**
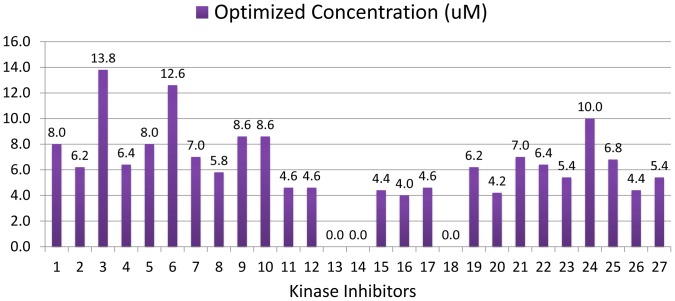
Optimal concentration of each kinase inhibitor for PC9 cancer cell line treatment.

According to the optimal concentration defined above, each kinase inhibitor in the KINOMEscan database can be tested by our system model to evaluate its capability of controlling the proliferation of PC9 cancer cells while avoiding severe damage to liver cells. In this paper, 27 kinase inhibitors have been screened and the optimal concentrations for these kinase inhibitors were shown in Figure 16. Obviously, three kinase inhibitors with 0 uM optimal concentration should be discarded since they have less effect on PC9 cancer cells. In the remaining candidates, we selected the six kinase inhibitors which have the highest integrated effect indexes as listed in Table 3. In these kinase inhibitors, we can see that MLN8054 can produce a best effect index but needs higher concentration than PF02341066. Overall considering effect index, cancer cell number and optimal concentration, PF02341066 is suggested to be the best candidate for PC9 cancer cell treatment, since it has the similar effect with MLN8054 while requiring a lower concentration. In fact, PF02341066, known as Crizotinib, has been approved by the U.S. Food and Drug Administration for the treatment of ALK-rearranged NSCLC in August 2011 [27], which supports the prediction from our system approach.

**Table 3:** Six kinase inhibitors with highest effect index for PC9 cell line.

Kinase Inhibitor	MLN8054	PF02341066	JWE-035	GSK461364	AZD-6482	NU7441
Effect Index	0.8070	0.8070	0.8065	0.8063	0.8055	0.8053
Cancer Cell Number	3.4931	3.4929	3.4928	3.4927	3.5185	3.5269
Optimal Concentration	7.0	4.6	6.8	8.6	4.6	6.2

### Evaluations of Kinase Inhibitor Combinations

**Figure 17:**
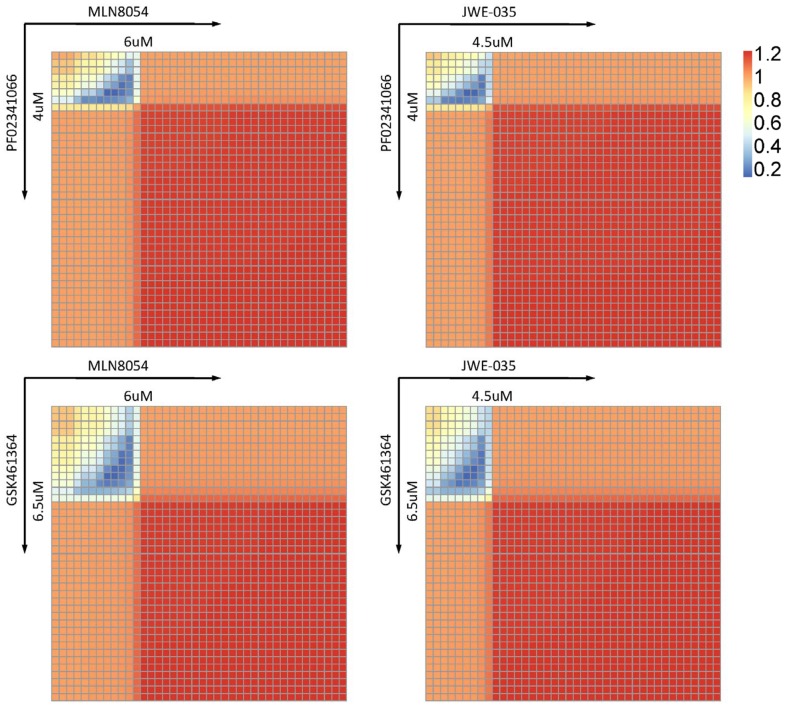
Combination effect of different kinase inhibitors. The combination effect is: I) synergy, if *CI_Bliss_*(*x*,*y*)<1. II) additivity, if *CI_Bliss_*(*x*,*y*) = 1. III) antagonism, if *CI_Bliss_*(*x*,*y*)>1.

It is well known that, for drug combination, two drugs working together may produce an effect greater than the expected combined effect of the same agents used separately. This case is known as synergic combination. Otherwise, we call the output of combination case as additive effect which produce equivalent effect or antagonism which produce less effect. In addition, different ratio combinations of dose for the same two drugs sometimes can produce totally different effects, such as one combination is synergistic while another is antagonistic. Therefore, it is also significant to predict the synergy combinations of dose ratios using our mathematical model. Although a number of available mathematical combination indexes can be used to assess the effect of drug combination, we prefer to choose Bliss independence [28], because it is not only a famous synergy quantification method but also convenient for calculation in our system model. According to this Bliss combination index *CI_Bliss_*(*x*,*y*), the combination effect can be considered as synergy if *CI_Bliss_*(*x*,*y*)<1, additivity if *CI_Bliss_*(*x*,*y*) = 1 and antagonism if *CI_Bliss_*(*x*,*y*)>1. Based on the prediction of integrated effect profiles for the kinase inhibitors in the KINOMEscan database, we choose the four best kinase inhibitors listed in Table 3 to analyse the combination effect of each two in PC9 cancer cell treatment. The simulation results for heatmaps of Bliss combination index are shown in Figure 17. It can be seen that all of them show the similar pattern which separates the synergetic, additive and antagonistic regions by two distinct and straight boundaries. Each boundary can correspond to a threshold for the kinase inhibitor, such as 4 uM for PF02341066 and 6 uM for MLN8054. When the concentrations of both combined kinase inhibitors are lower than their thresholds, the dose combinations will produce a synergetic effect. When only one kinase inhibitor achieves the concentration higher than its threshold, the combination effect is additive. If both the kinase inhibitors achieve the concentrations higher than their thresholds, they show antagonistic property. It is remarkable that the threshold for each kinase inhibitor will not change in different combinations. As a result, if we have obtained the thresholds for two kinase inhibitors separately, it will be easy to infer the synergistic region for the combination of these two kinase inhibitors. The predicted synergistic regions for different combinations are potentially helpful to conduct the clinical trials for drug combination.

## Discussion

Drug effect on the human body during the cancer treatment is not only the alteration of disease progression, but also the damage to the functions of different organs. Although, different drugs, such as the kinase inhibitors considered in this work, can target different proteins to inhibit the cancer cell proliferation, few detailed drug side effect profiles have been involved in literatures. In order to account for both therapeutic effect and side effect of kinase inhibitors, we focused on their effect on cancer cells as well as liver cells, since the liver plays a central role in transforming and clearing chemicals and is susceptible to the toxicity from these agents. In this paper, a system approach involving cancer cell specific pathway and primary human hepatocytes specific pathway was developed by integrating KINOMEscan data, proliferation/mitosis imaging data and cue signal response data in LINCS database.

The established PC9 cancer cell specific pathway model indicates the deficiency of two checkpoint related links which can result in abnormal expansion of cell number. This kind of signature from cancer cell specific pathway model can be used as a potential feature to evaluate the risk of cancer development. Sensitivity analysis of PC9 cancer specific pathway reveals several critical cascades on modulating the proliferation process of the cancer cell. These cascades are a part of PI3K/AKT pathway, which is associated with cell survival and proliferation. In many human cancer and tumour cells, this pathway is overactive reducing apoptosis and allowing proliferation. Our mathematical model also confirms the important role of PI3K/AKT pathway in PC9 cancer cell. As consistent with previous study [23], [24], targeting the proteins in PI3K/AKT pathway is suggested to be a therapeutic approach for PC9 cancer.

In order to assess the integrated effect of kinase inhibitors on both cancer cells and liver cells, a normalized effect index is defined based on the number of cancer cells and the levels of several selected proteins in liver cells after treatment. According to the results obtained from simulation, an optimal concentration can be suggested for each kinase inhibitor. When overall considering the integrated effect index, cancer cell number and optimal concentration, PF02341066 is screened out to be the proper kinase inhibitor for PC9 cancer treatment while avoiding damage to primary human hepatocytes from 27 candidates. Furthermore, the simulation result from drug combination analysis shows that the synergistic effect is located in rectangular region. It is worth noting that the boundary of this rectangular region can be determined by a threshold for each kinase inhibitor. This threshold seems to be an inherent property of the kinase inhibitor and will not change no matter what another kinase inhibitor is in the drug combination. Based upon this feature, it will be straightforward to predict the synergistic regions of different combinations once the threshold for each kinase inhibitor has been already available.

In this work, binding affinity prediction is an important input of our system model. However, it is only based on a single point form KINOMEscan data and a simple assumption for our simulation. Such kind of prediction may be not accurate enough because of the lack of binding information. In the future, more KINOMEscan data on different concentration levels of the kinase inhibitors will help to improve the simulation results and provide significant information for optimizing drug concentration.

Although our model is established based on the data of PC9 cell line in this paper, this system approach will be easy to generate specific model for other cancer cell using corresponding imaging data. Thus, we provide an online tool named KIEP (Kinase Inhibitor Effect Prediction) for users to test a kinase inhibitor effect on the cancer cells they concern *in* silico (http://ctsb.is.wfubmc.edu/itNETZ/KIEP.html). As we can expect, this model has the potential capability of predicting drug effect on different cancer cells and liver cells to conduct the drug design or combination before clinical trials.

## Materials and Methods

### KINOMEscan Assay and Binding Affinity Prediction

KINOMEscan platform is based on a competition binding assay that is run for a compound of interest against each of a panel of over 400 kinases. This assay has three components: a kinase-tagged phage, a test compound, and an immobilized ligand that the compound competes with to displace the kinase. The amount of kinase bound to the immobilized ligand is determined using quantitative PCR of the DNA tag. The readout from assay is “percent of control”, where the control is DMSO and where a 100% result means no inhibition of the kinase by the test compound.

In order to extend the binding affinity from KINOMEscan data at 10 uM in LINCS database, a sigmoid function was adopted to represent the relationship between “percent of control” and the kinase inhibitor concentration, which was expressed as
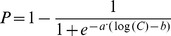
(1)where *P* is the “percent of control” and *C* is the kinase inhibitor concentration; a and b are two parameters which determine the shape of the function. In order to obtain the values of unknown parameters a and b for each kinase bound by its inhibitor, at least two data points are needed. Besides the KINOMEscan data at 10 uM concentration level, we assumed that the “percent of control” under extremely low concentration (ELC) of kinase inhibitor is 99%, as shown in Figure 3. Since few binding events occur under low concentration condition, this assumption is reasonable and acceptable. Based on two points, unknown parameters a and b can be acquired. Then, we can use Equ (1) to predict the “percent of control” values for any given concentration level.

### Cancer Cell Line Proliferation/Mitosis Imaging Assay

In LINCS project, dose response of eight kinase inhibitors in seventeen human cancer cell lines was tested to determine the effect on cell proliferation and mitosis. In these assays, each kinase inhibitor was diluted into twelve different concentrations with two duplicates. Before the exposition, Hoechst, EdU and anti-MPM2 primary antibody were added for identification of all nuclei, actively proliferating cells and mitotic cells, respectively [29]–[31]. Then at 24, 48 and 72 hours after exposition, cells were imaged using the ImageXpress Micro screening microscope. Each acquired image has three channels called DAPI, FITC and Texas Red which can record the signal intensities of Hoechst, EdU and anti-MPM2 primary antibody, respectively. Finally, image analysis using a customized Matlab program [32] was performed on these channels to report five readouts as listed in Table 2.

### Mathematical Model for Cancer Cell Pathway

Based on the cell cycle related pathway, a mathematical model was developed using hill function to represent the activating or inhibiting profile of a protein by its upstream protein. In biochemistry, the binding of a ligand to a macromolecule is often enhanced if there are already other ligands present on the same macromolecule, known as cooperative binding. Analogously, protein activation is also a kind of binding process. The Hill function has the capability of providing a way to quantify this effect. Thus, for each protein in the pathway, its variation was quantified by the summation of a series of hill functions and a degradation term, which can be expressed as
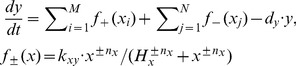
(2)where *y* is the concentration of activated proteins; 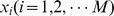 are the *i*th protein which activates protein y, while 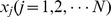 are the *j*th proteins which inhibits protein *y*; 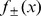 is the activating profile (+) or inhibiting profile (−) caused by protein *x*, respectively; 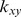 is activating rate (+) or inhibiting rate (−); 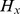 is the microscopic dissociation constant and 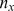 is the Hill coefficient; 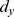 is the degradation rate of protein *y*. There are totally 21 ODEs and 151 parameters in this pathway model (detailed equations are listed in Text S1.).

In this paper, we used the proliferation/mitotic data of PC9 cell line treated by GW843682 to establish the PC9 cell line specific pathway. In order to train the mathematical model using this kind of data, it is necessary to convert the output of the model into the readouts from the assay. According to the readouts listed in Table 2, we generated four variables based on the pathway output G1, S, G2 and M, which can be expressed as
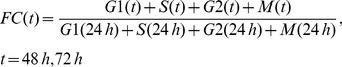
(3)

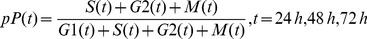
(4)

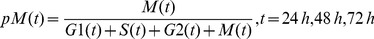
(5)

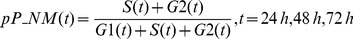
(6)where *t* is the time point, such as 48 h and 72 h; *FC*(*t*) is the fold change of cell number while compared to the cell number at 24 h; *pP*(*t*) and *pM*(*t*) are the percentage of actively proliferating cells and mitotic cells, respectively; *pP_NM*(*t*) is the percentage of actively proliferating cells within non-mitotic population. From these definitions, we can obtain 11 values for each test with a given concentration level.

### Mathematical Model for Primary Human Hepatocyte Pathway

Based on primary human hepatocyte pathway, mass action law was employed to build an ordinary differential equation for each protein, which can be expressed as
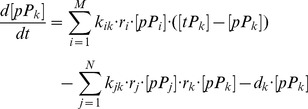
(7)where 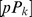 and 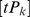 are the phosphorylated and total amount of *k*th protein. 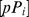 and 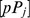 are the phosphorylated proteins which can activate and inhibit the phosphorylation of *k*th protein with the reaction rate 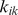 and 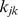, respectively. 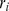 is binding rate of drug to *i*th phosphorylated protein, which can be obtained based on the KINOMEscan data. 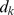 is the degradation rate of *k*th phosphorylated protein. There were totally 52 ODEs and 83 parameters in this primary human hepatocyte pathway model (detailed equations are listed in Text S2).

### Parameter Estimation

In order to produce an optimized fitting simulation results to the corresponding experimental data, we minimize the following objective function to obtain the estimate for all parameters.
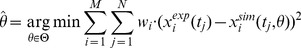
(8)where 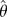 is estimate of the parameters and 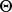 is the parameter space for 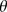; 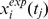 and 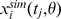 represent the observation from the assay and the calculated value from simulation with parameters 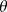 at time point 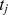, respectively; 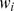 is the weight to rescale the errors from different measurements. Genetic algorithm [33] is adopted as the search algorithm to minimize the objective function. Due to the huge parameter space in this model, a two stage approach was used to obtain a more reliable estimate for the parameters. In the first stage called model selection, we repeated the search algorithm fifty times within this huge parameter space and chose the best estimate which generated the minimum error between results from simulation and assay. Then, based on the parameters which were zero in the best estimate, the model can be simplified by removing the links or degenerating the hill functions. In the second stage called parameter refinement, the non-zero parameters were refined by repeating the search algorithm in a reduced parameter space. After these two stages, an optimized combination of parameters can be located in the parameter space to represent the experimental data felicitously.

### Parameter Sensitivity Analysis

Parameter sensitivity analysis is used to examine whether the system is preserved to the modest parameter changes and quantitatively explore the sensitive parameters. In this work, local parameter sensitivity analysis is employed to study the relationship between cancer cell number and the perturbation on each parameter value. The sensitivity coefficient (S) is calculated according to the following formula:
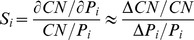
(9)where *CN* is the cell number at 72 h after treatment; *P_i_* is the *i*th estimated parameters in the system and Δ*P_i_* is a small change of the corresponding parameter. In this work, each parameter is increased by 2% of its estimated value for investigating the corresponding change of cell number.

### Integrated Effect Index based on Therapeutic and Side Effect

For therapeutic effect, cancer cell number is the only critical factor we would like to control in the pathway model while there is not a unique factor representing the side effect. For simplification, we first choose ERK, p70S6K, CREB, cJun and NFkB as the proteins representing the side effect, since these proteins are directly related to the protein synthesis or cell survival in our pathway model. Then the levels of these five proteins were divided by the cancer cell number to generate five ratios, denoted by *r_ERK_*, *r_p70S6K_*, *r_CREB_*, *r_cJun_* and *r_NFkB_*. In order to integrate these ratios into a novel factor, we normalize them using the formula expressed as
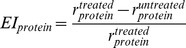
(10)where *EI_protein_* is the normalized effect index based on each protein, such as ERK or p70S6K; 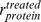 and 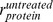 are the ratios obtained from the kinase inhibitor treated and untreated condition. Finally, the five normalized ratios were averaged into a novel factor denoted by *EI_mean_*, which can be used to assess the integrated effect of a kinase inhibitor.

### Bliss Combination Index

For kinase inhibitor 1 and 2 in this study, given the system input with dose combination (*x*,*y*), the corresponding Bliss combination index *CI_Bliss_* (*x*,*y*) is defined as follow
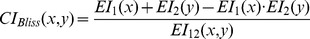
(11)where *EI*_1_(*x*) and *EI*_2_(*y*) are the integrated effect indexes for the single kinase inhibitor 1 with dose *x* and the single kinase inhibitor 2 with dose *y*, respectively; *EI*_12_(*x*,*y*) is the integrated effect index for the kinase inhibitor 1 and 2 combination with dose (*x*,*y*). Based on this Bliss combination index, the combination effect can be considered as synergy if *CI_Bliss_*(*x*,*y*)<1, additivity if *CI_Bliss_*(*x*,*y*) = 1 and antagonism if *CI_Bliss_*(*x*,*y*)>1.

## Supporting Information

Figure S1 **Comparison between simulation results and proliferation/mitosis data of PC-9 cell line.** MSE: Mean square error.
(DOCX)

Figure S2 **Sensitivity analysis of parameters in PC9 cell line pathway model.**
(DOCX)

Figure S3 **Simulation results from primary human hepatocyte pathway model trained by the cue signal response data.**
(DOCX)

Text S1 **Mathematical model for cell cycle related pathway.**
(DOCX)

Text S2 **Mathematical model for primary human hepatocyte pathway.**
(DOCX)
