# Supplementary material for: Systematically Studying Kinase Inhibitor Induced Signaling Network Signatures by Integrating Both Therapeutic and Side Effects
Source: PLoS One. 2013 Dec 5;8(12):e80832. doi: 10.1371/journal.pone.0080832 (PMC3855094; doi:10.1371/journal.pone.0080832)
Supplement: Figure S1 — Comparison between simulation results and proliferation/mitosis data of PC-9 cell line. MSE: Mean square error. (DOCX) [file pone.0080832.s001.docx]

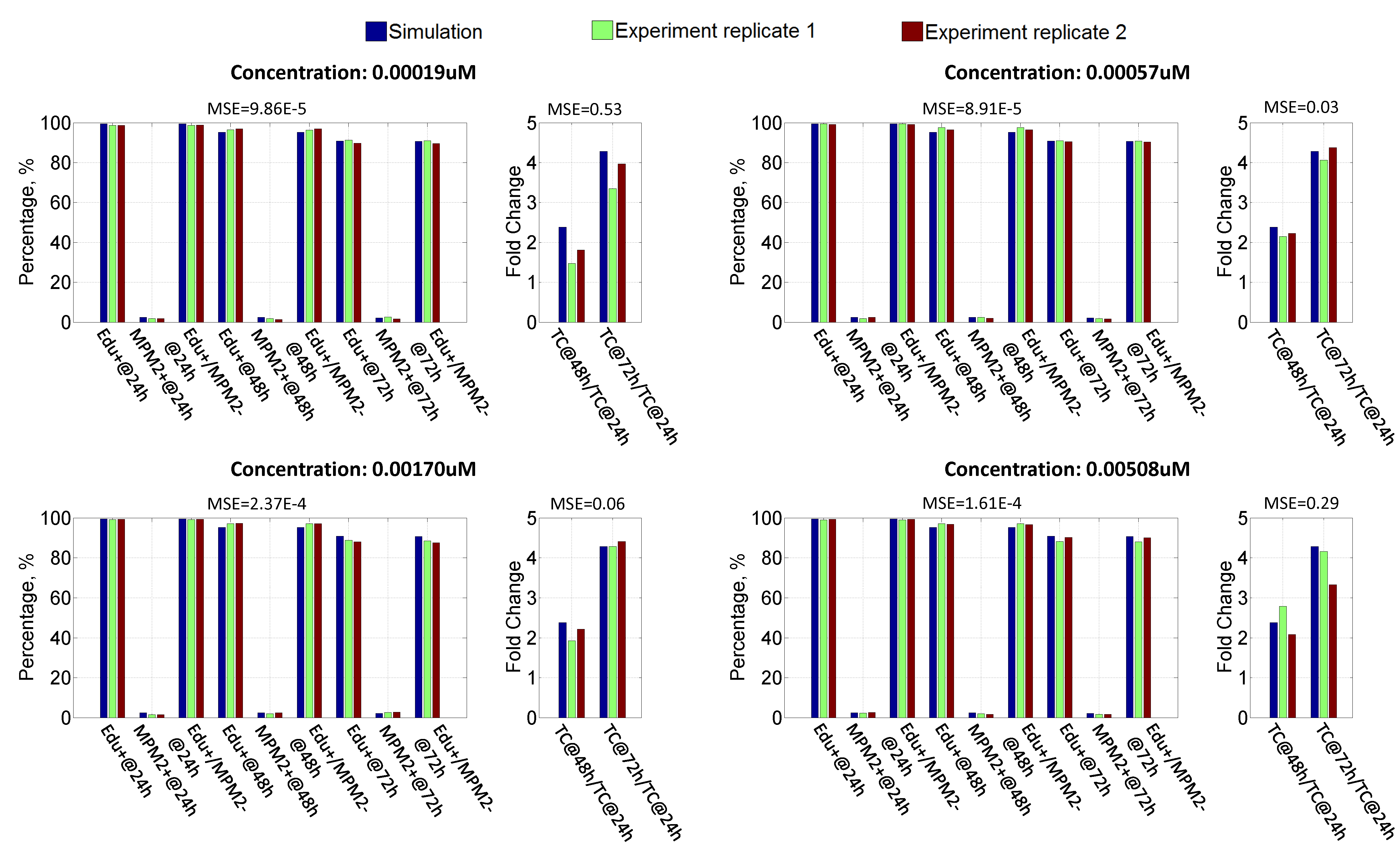

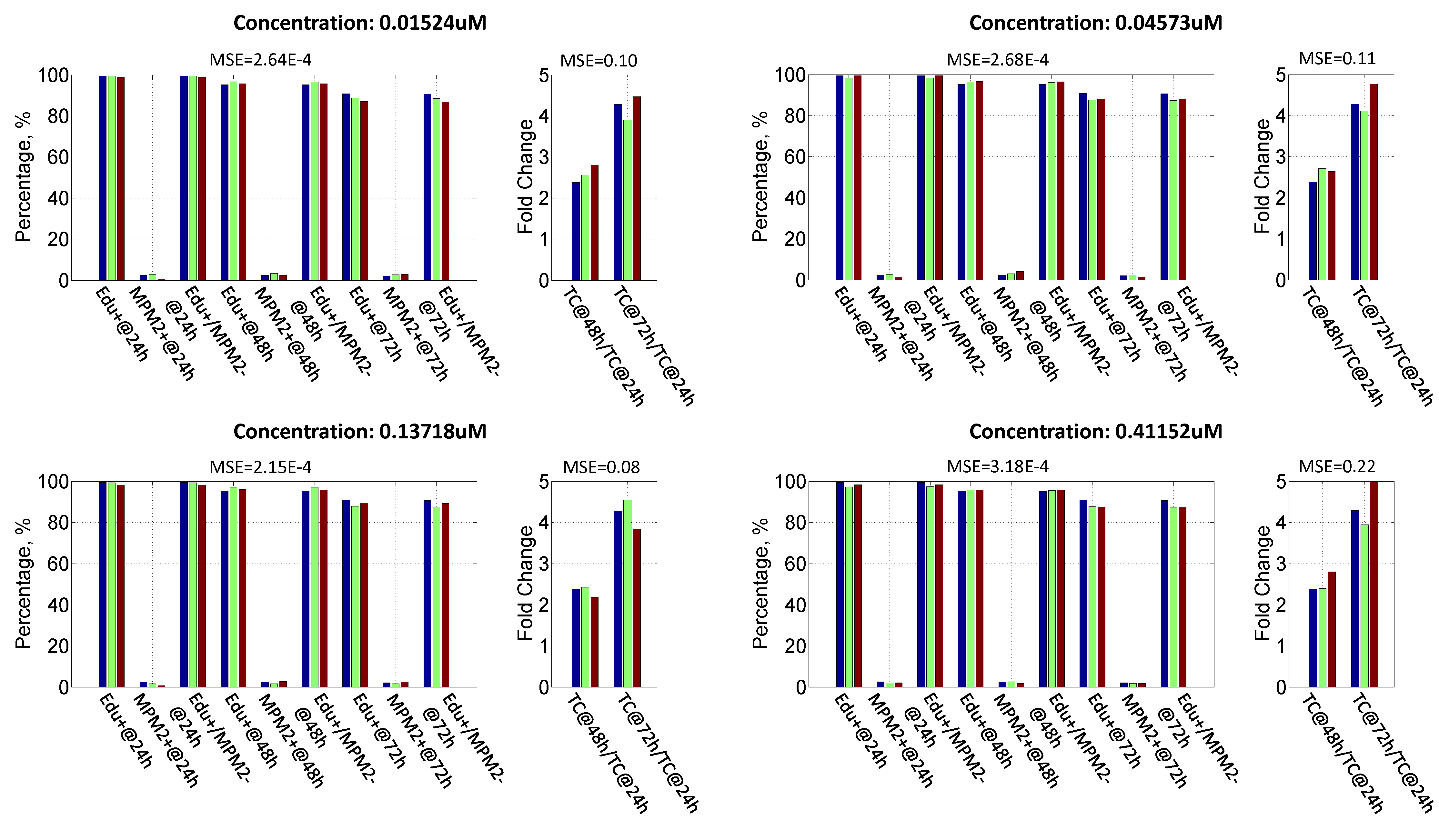


Figure S1 Comparison between simulation results and proliferation/mitosis data of PC-9 cell line.

MSE: Mean square error.
