# Supplementary figures and images for: Systematically Studying Kinase Inhibitor Induced Signaling Network Signatures by Integrating Both Therapeutic and Side Effects
Source: PLoS One. 2013 Dec 5;8(12):e80832. doi: 10.1371/journal.pone.0080832 (PMC3855094; doi:10.1371/journal.pone.0080832)

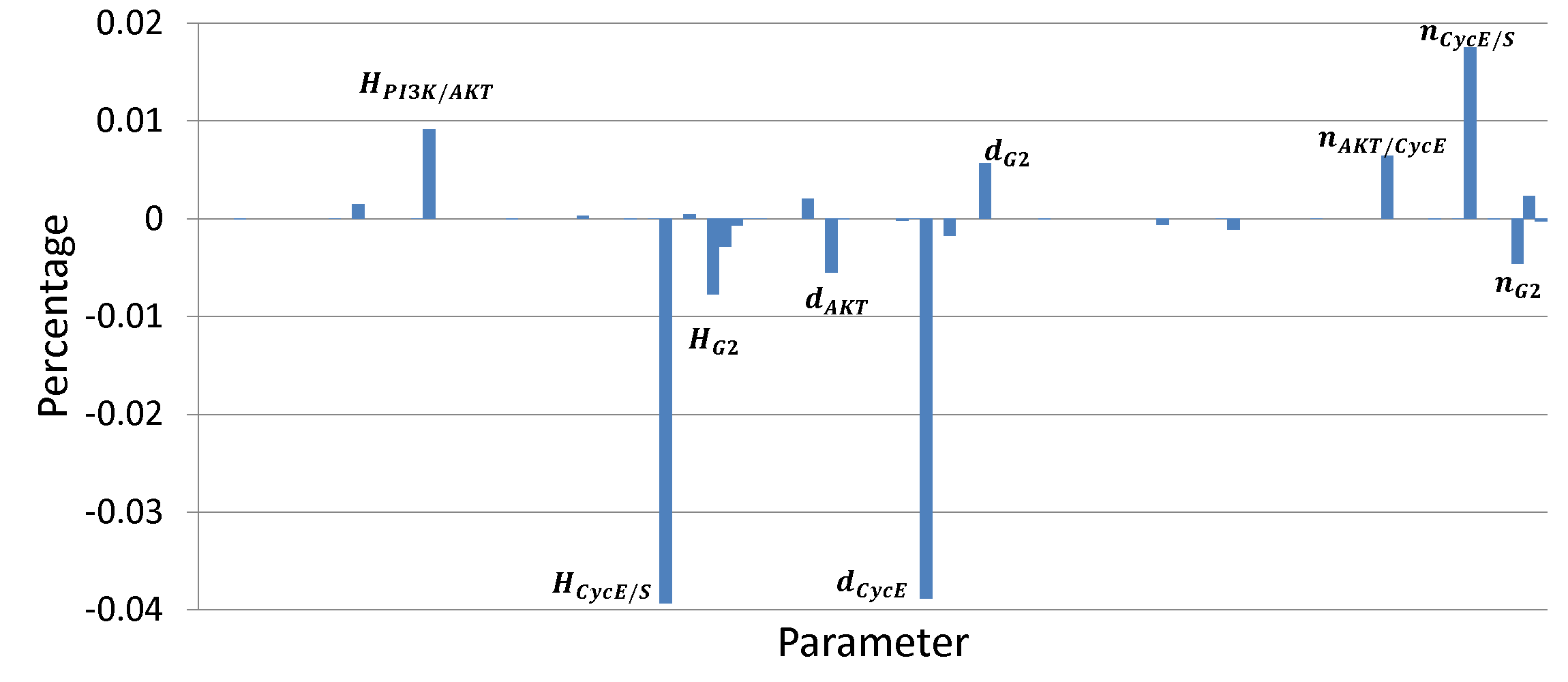


Figure S2 Sensitivity analysis of parameters in PC9 cell line pathway model.

Supplement: Figure S2 — Sensitivity analysis of parameters in PC9 cell line pathway model. (DOCX) [file pone.0080832.s002.docx]
