# Supplementary material for: Systematically Studying Kinase Inhibitor Induced Signaling Network Signatures by Integrating Both Therapeutic and Side Effects
Source: PLoS One. 2013 Dec 5;8(12):e80832. doi: 10.1371/journal.pone.0080832 (PMC3855094; doi:10.1371/journal.pone.0080832)
Supplement: Figure S3 — Simulation results from primary human hepatocyte pathway model trained by the cue signal response data. (DOCX) [file pone.0080832.s003.docx]

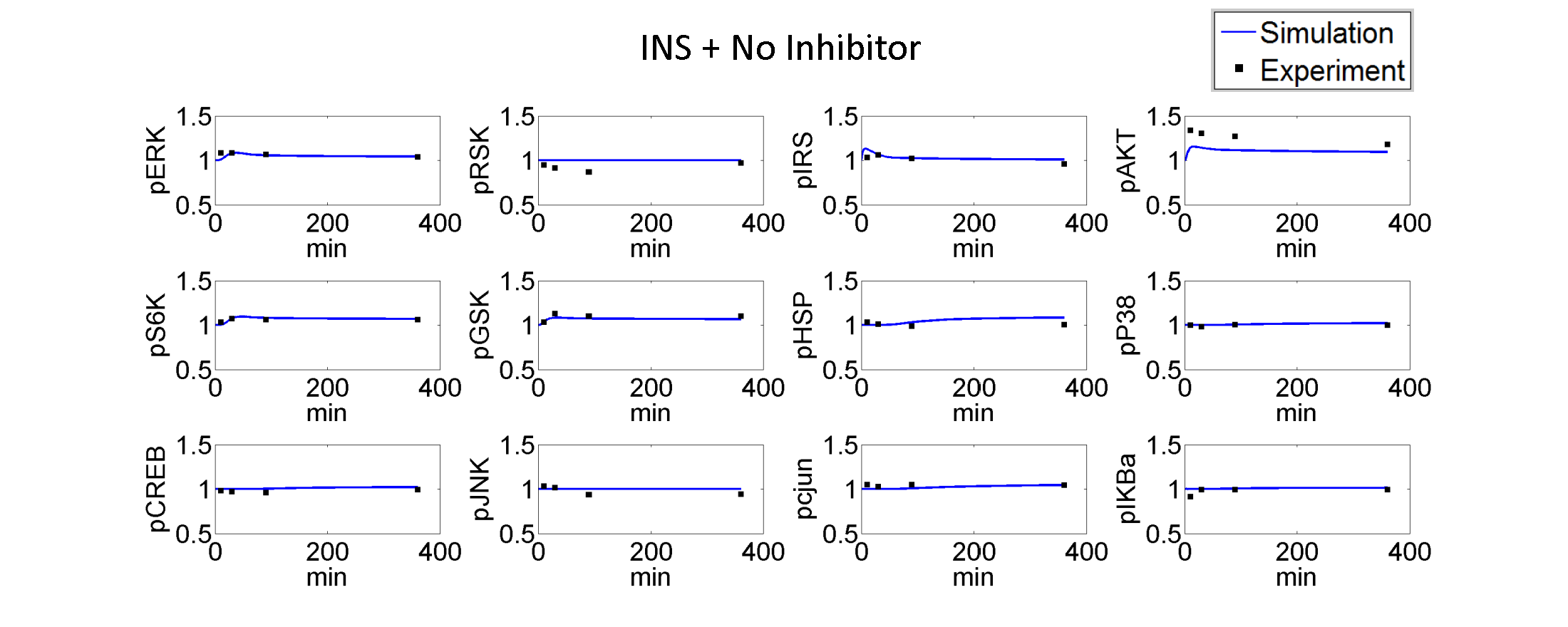

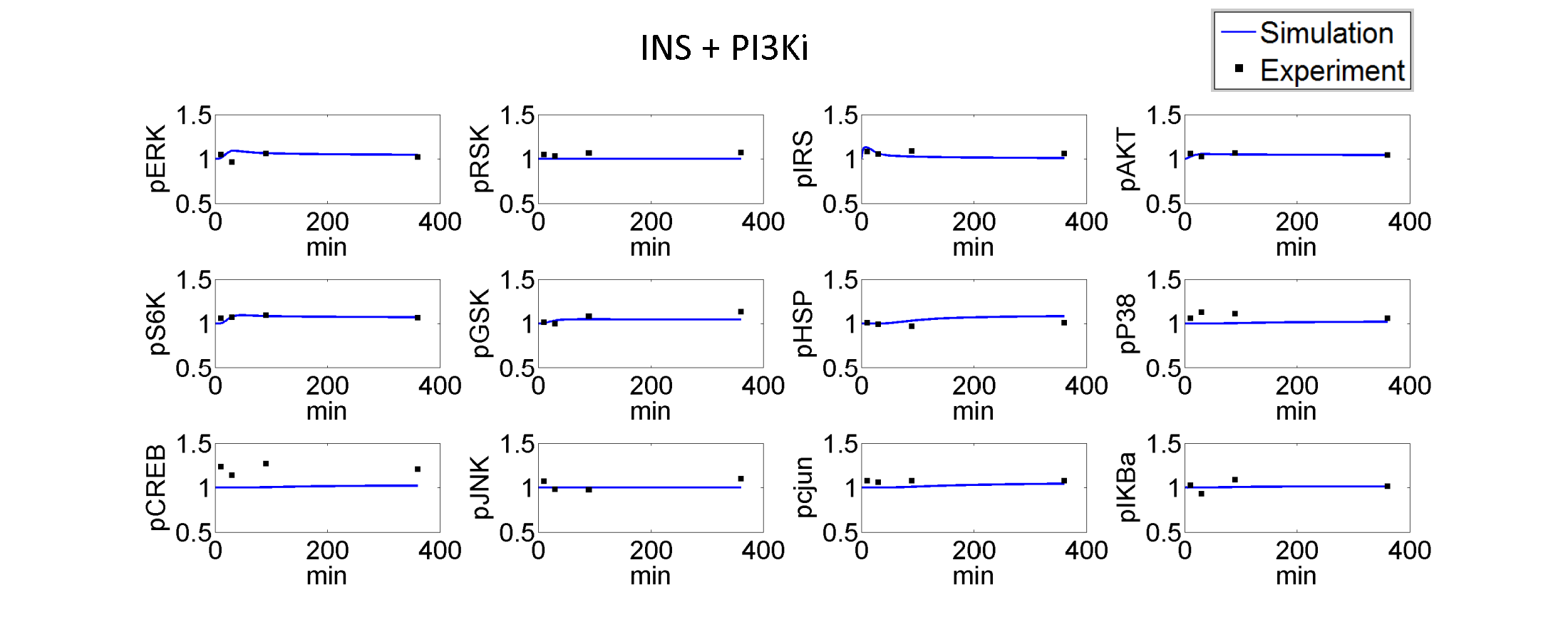

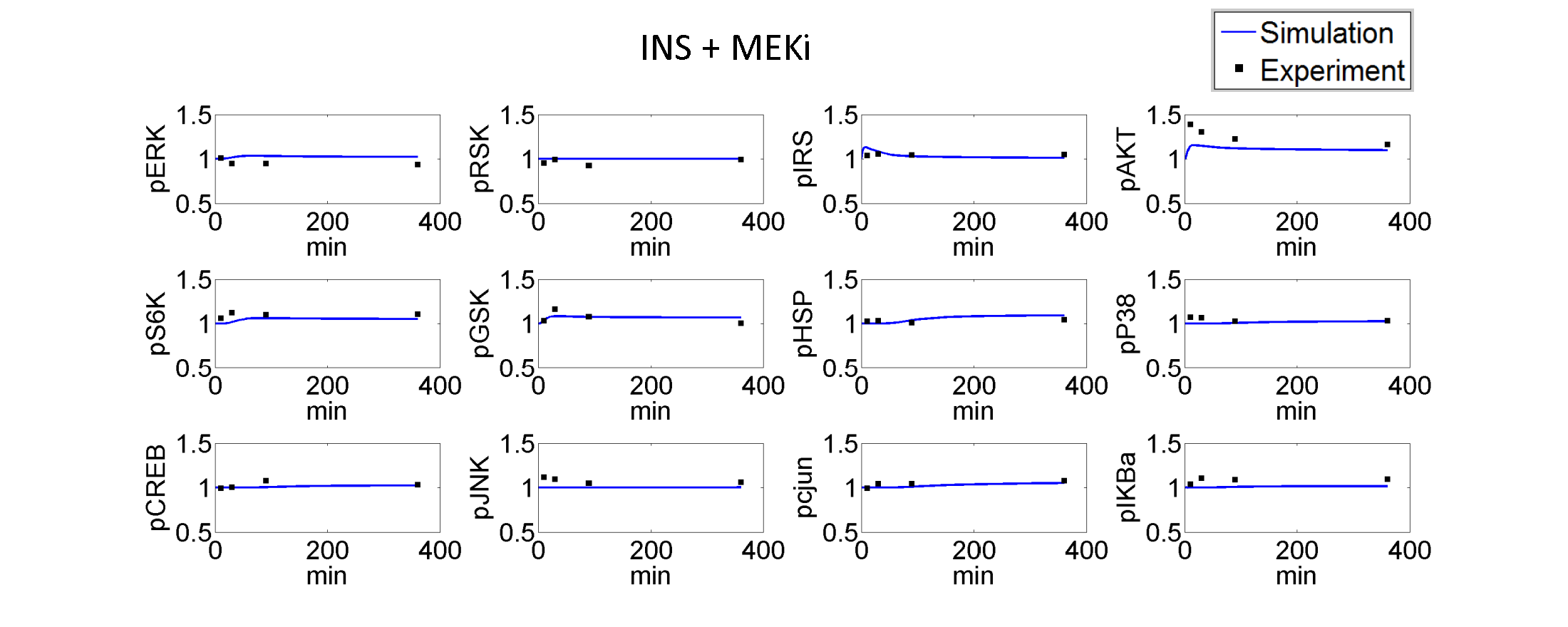


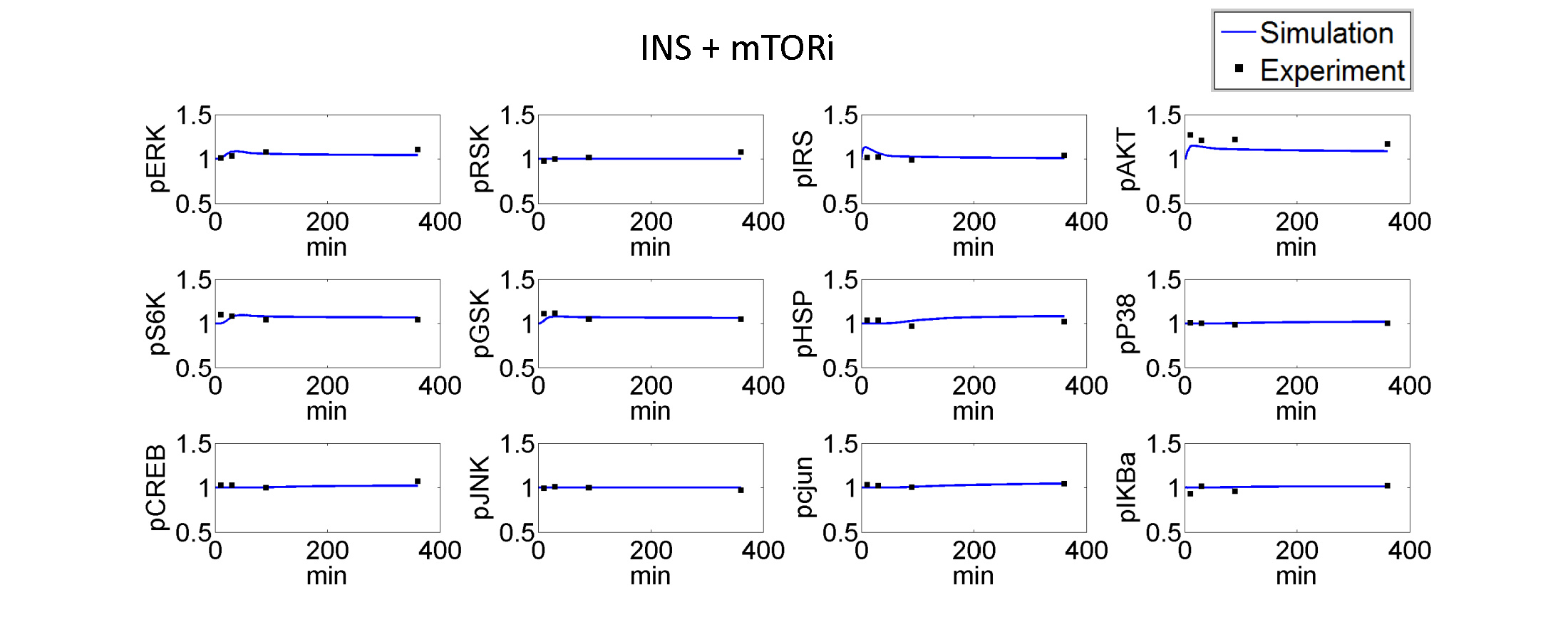

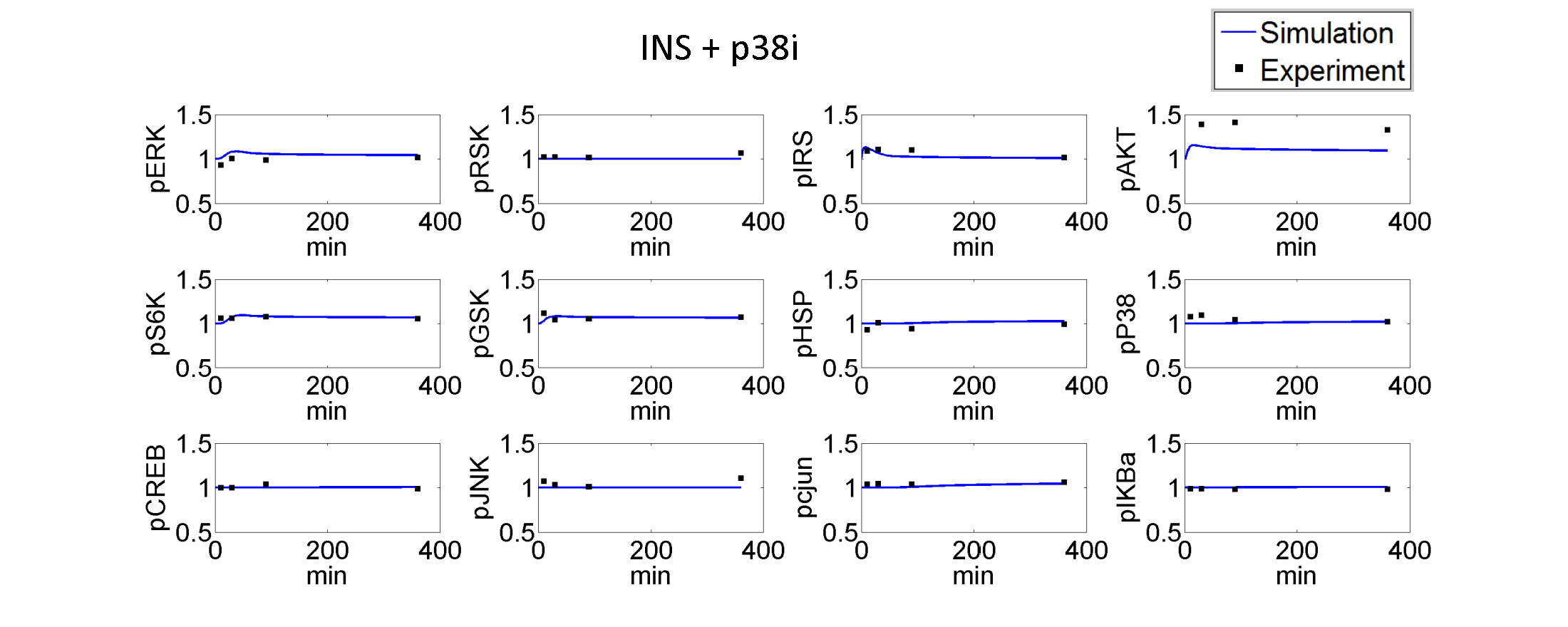

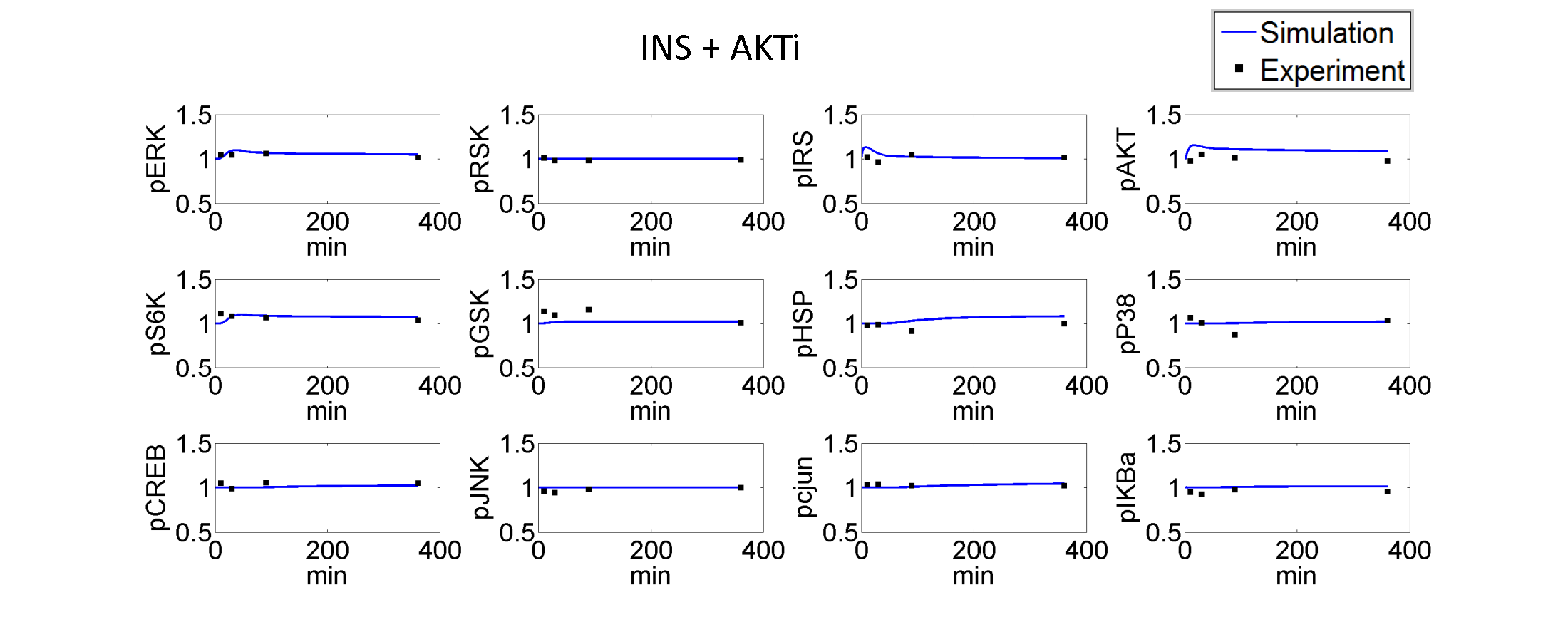


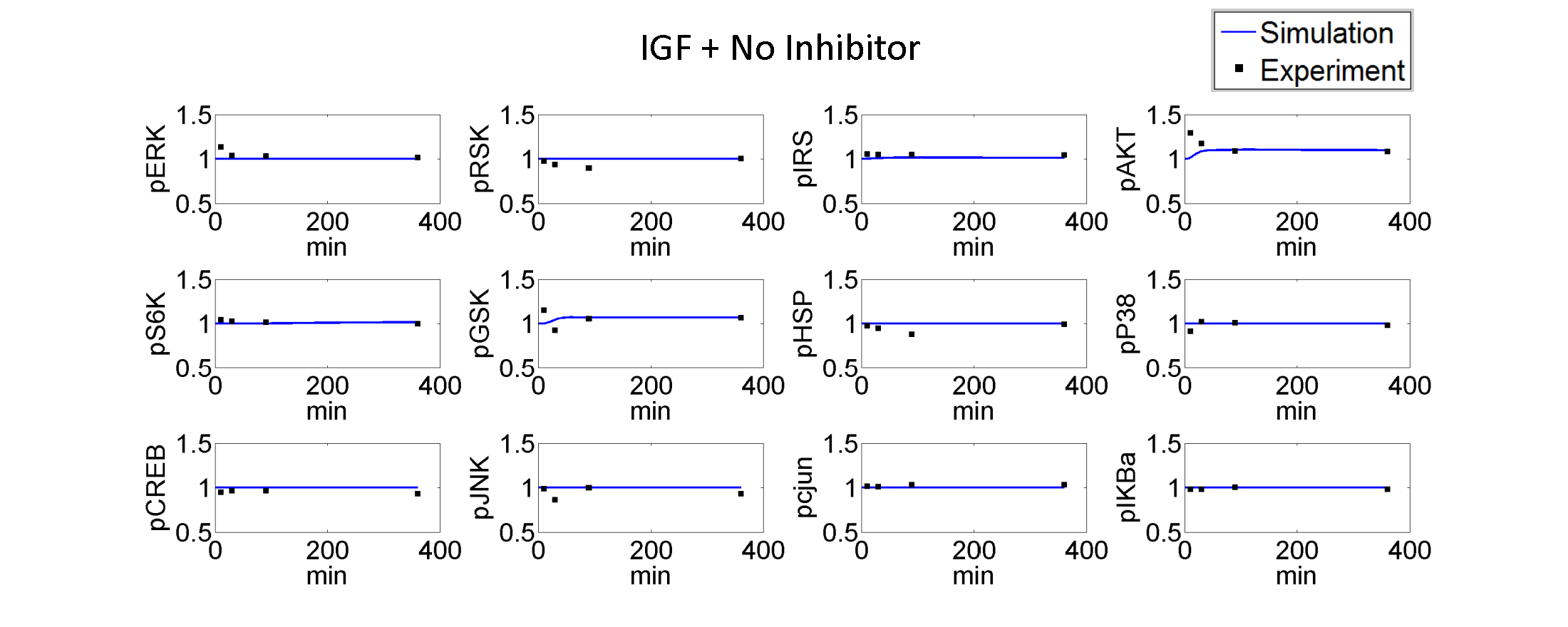

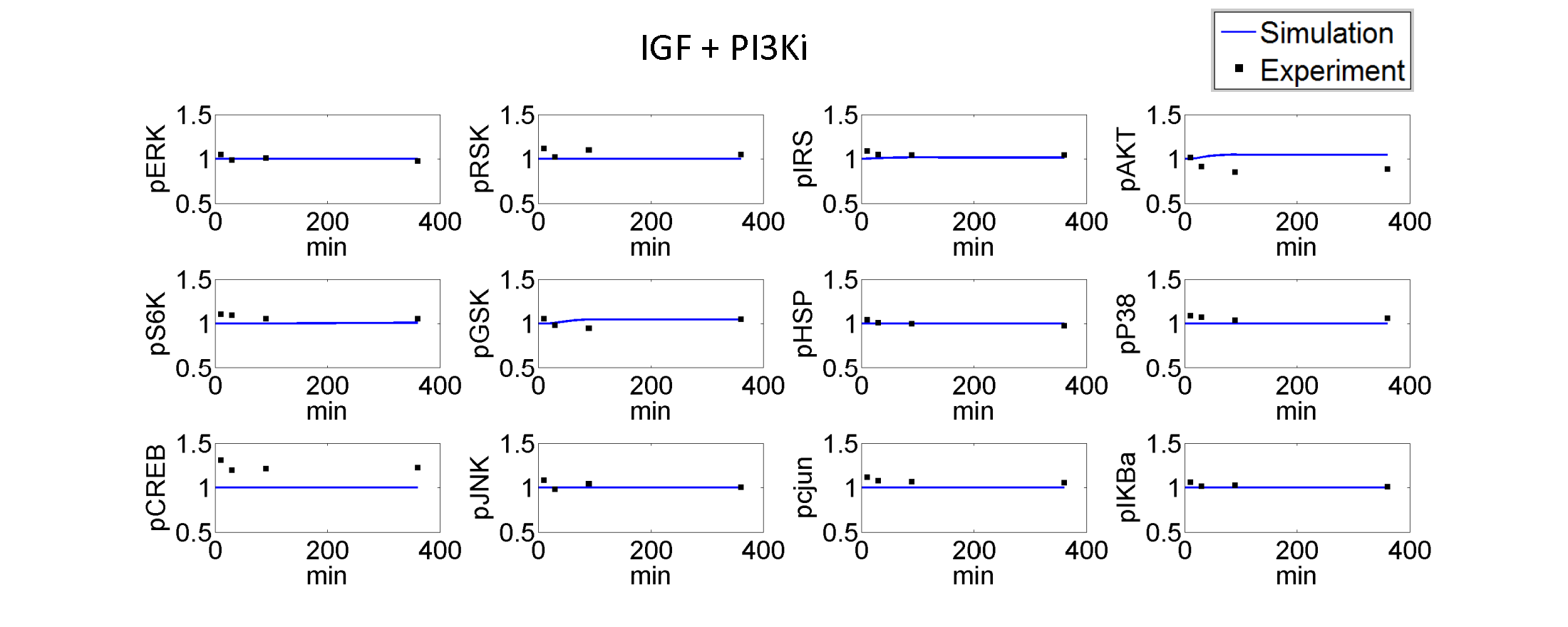

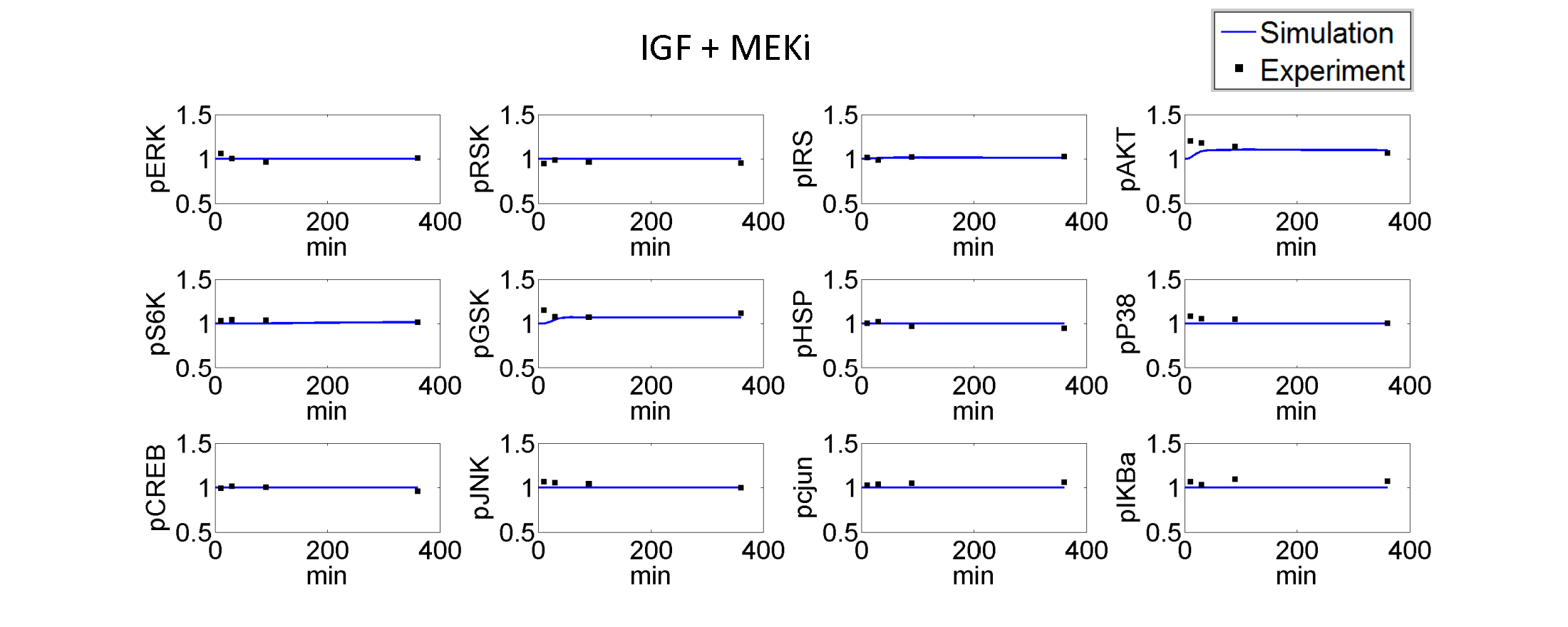


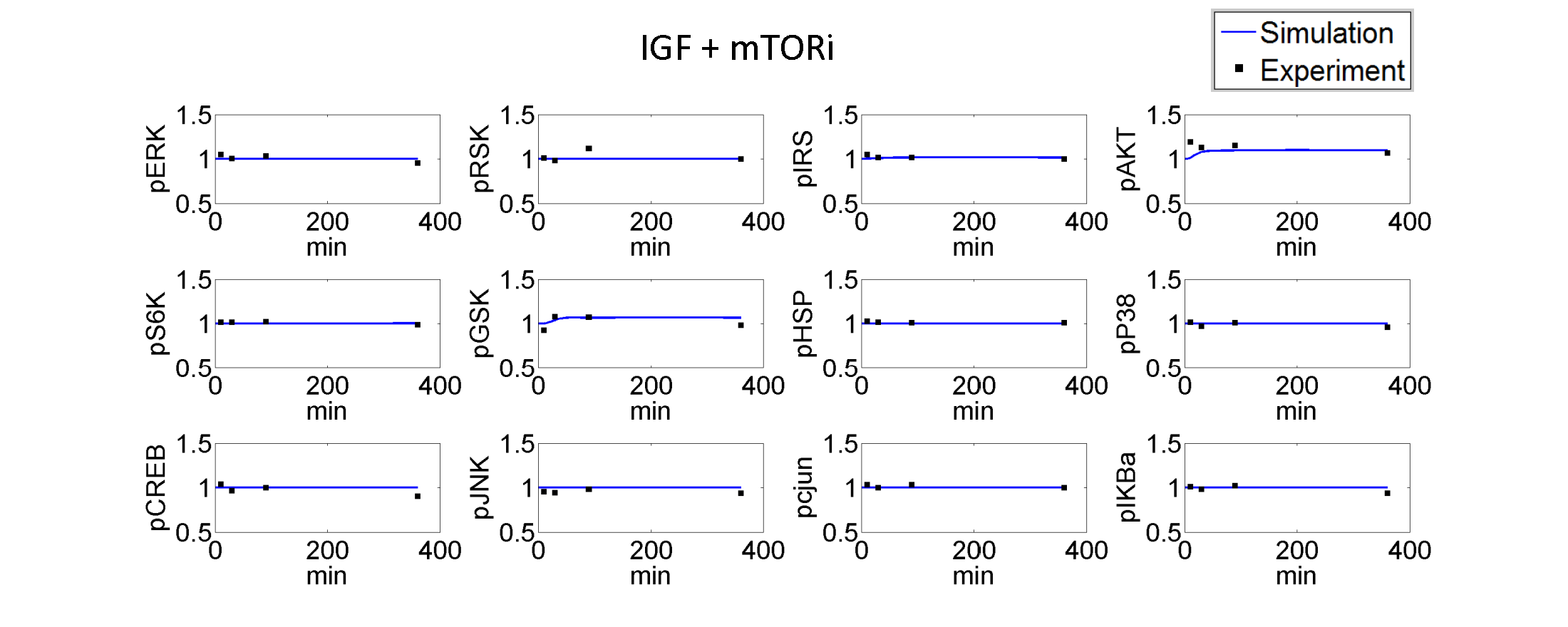

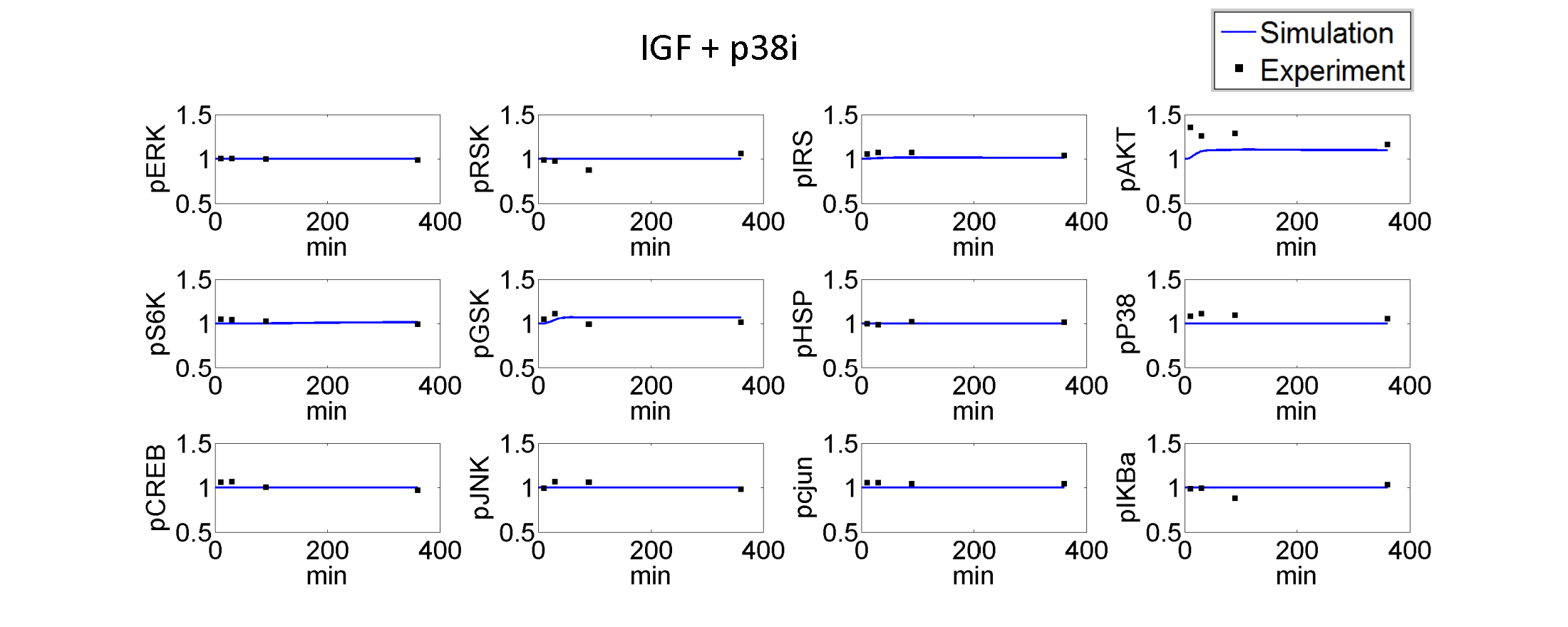

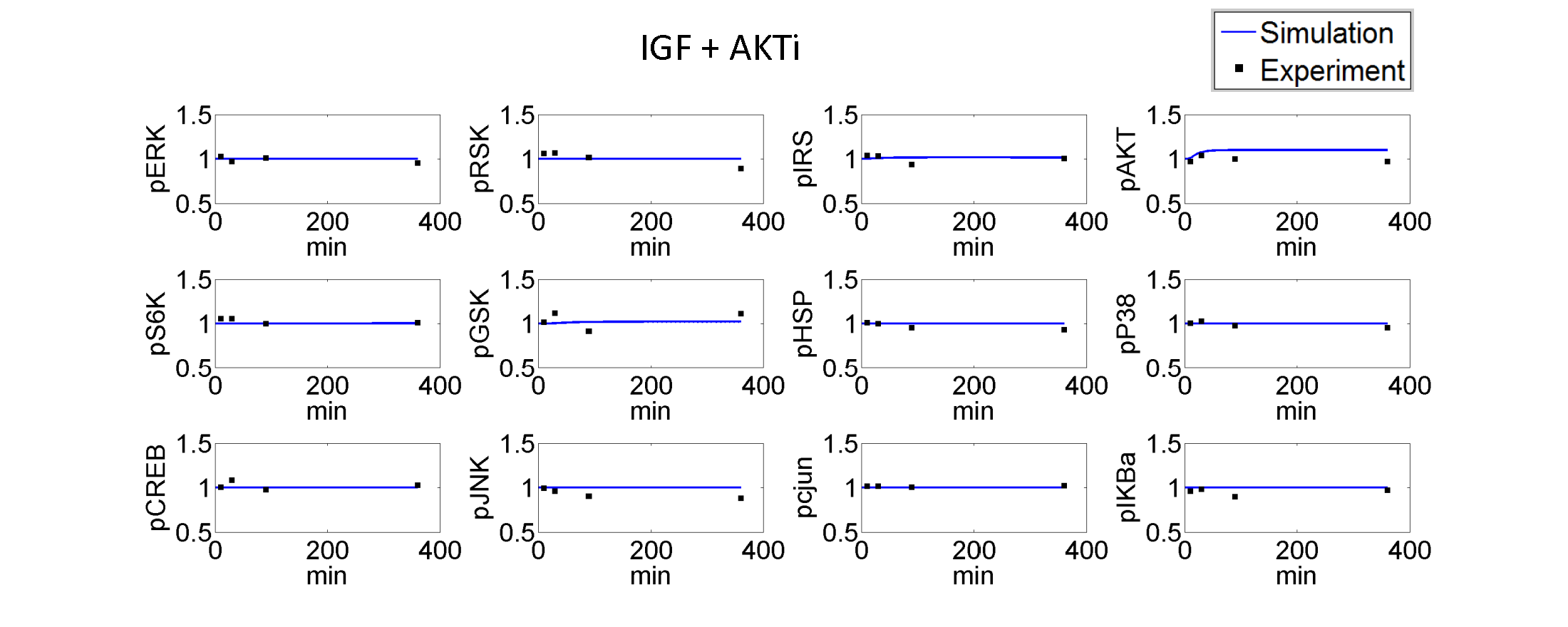


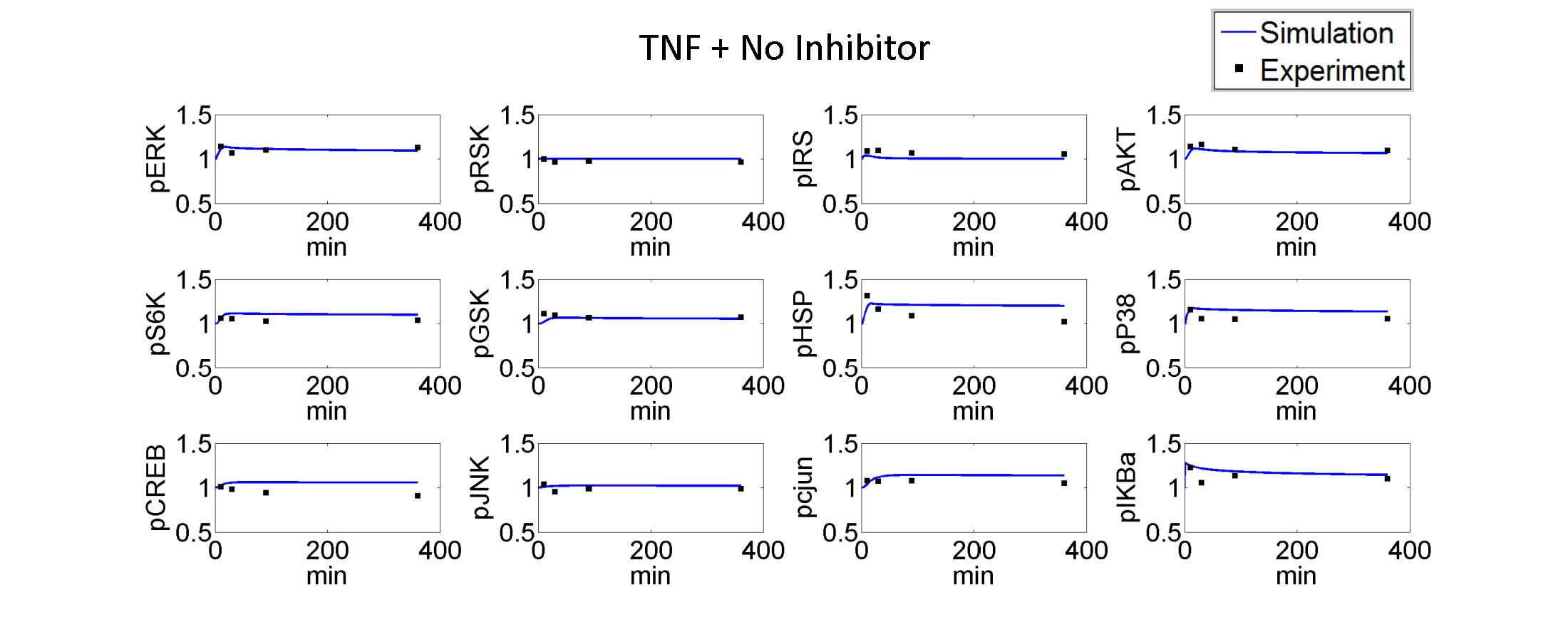

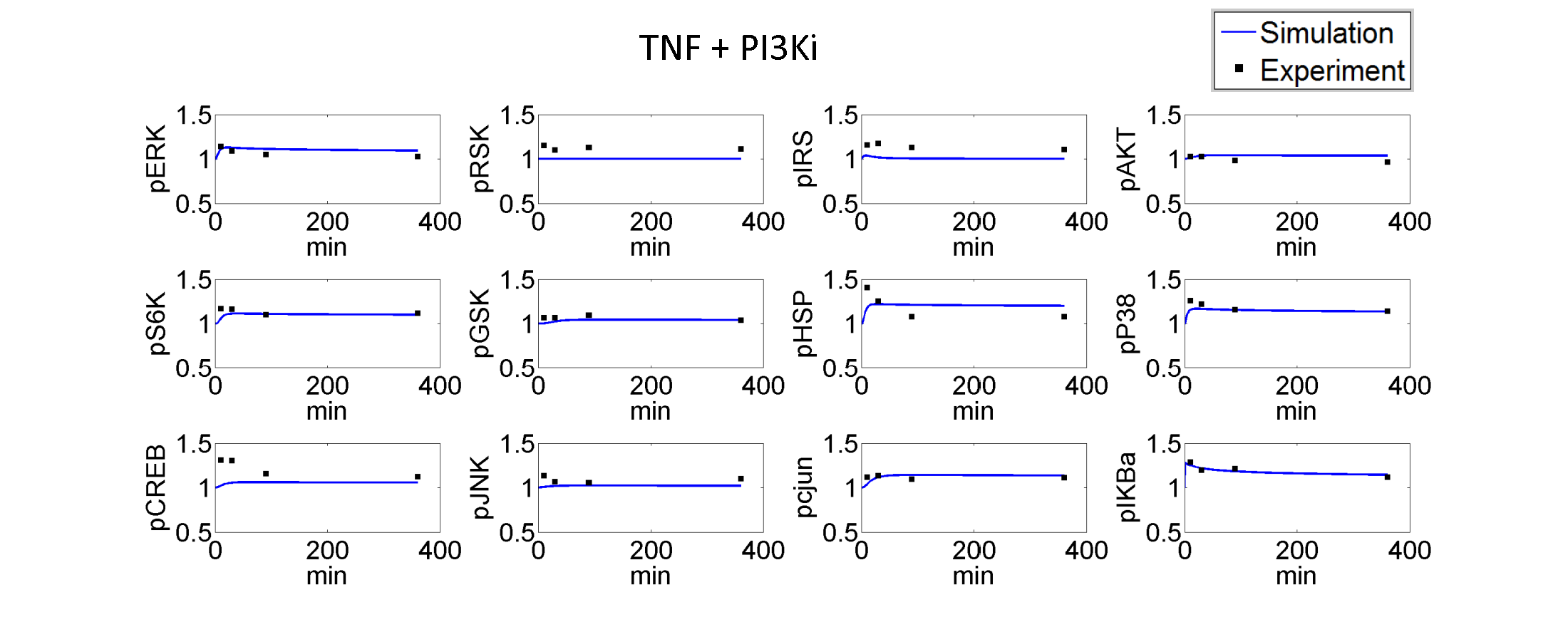

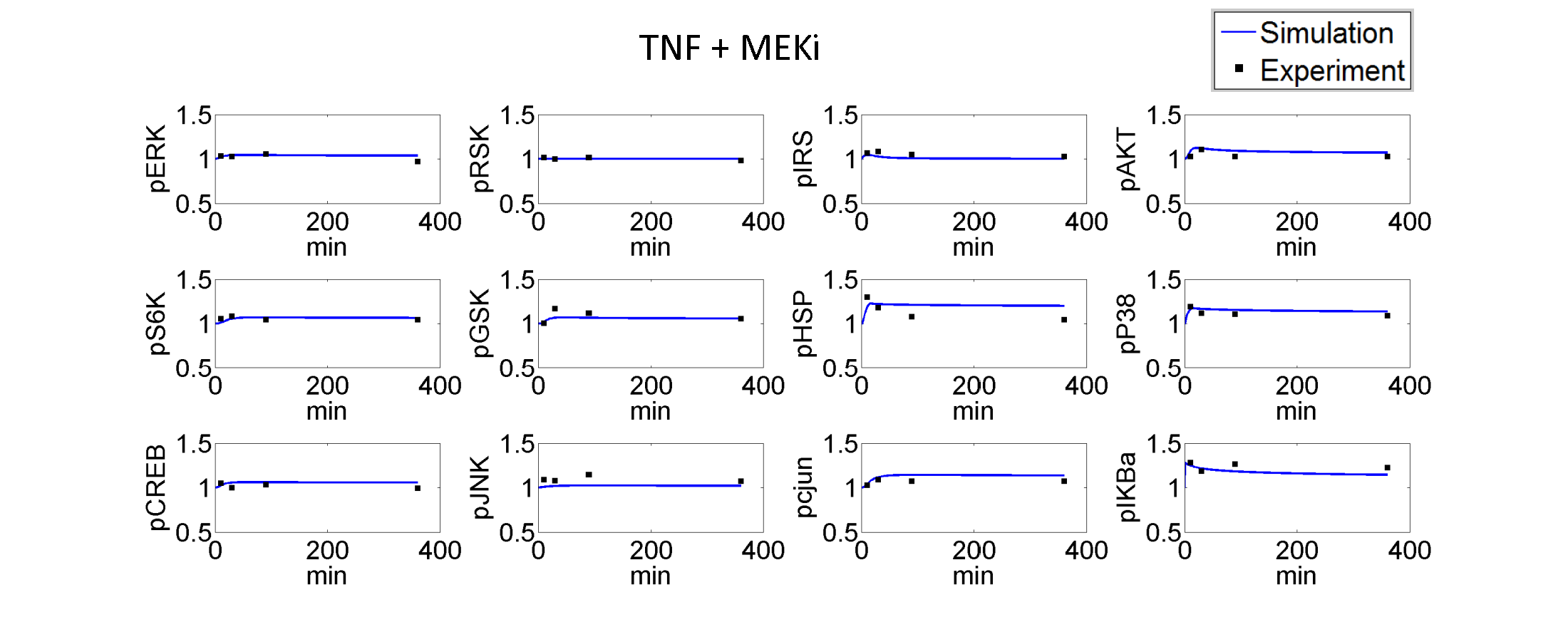


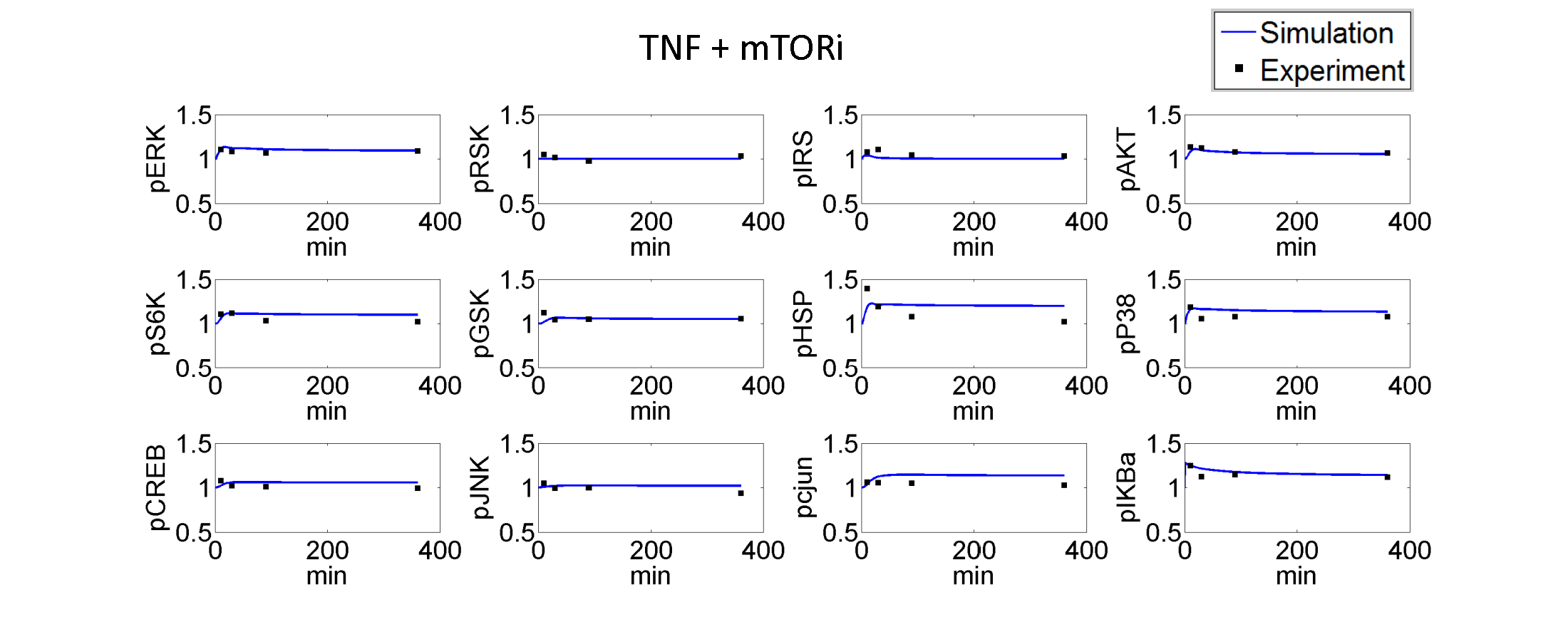

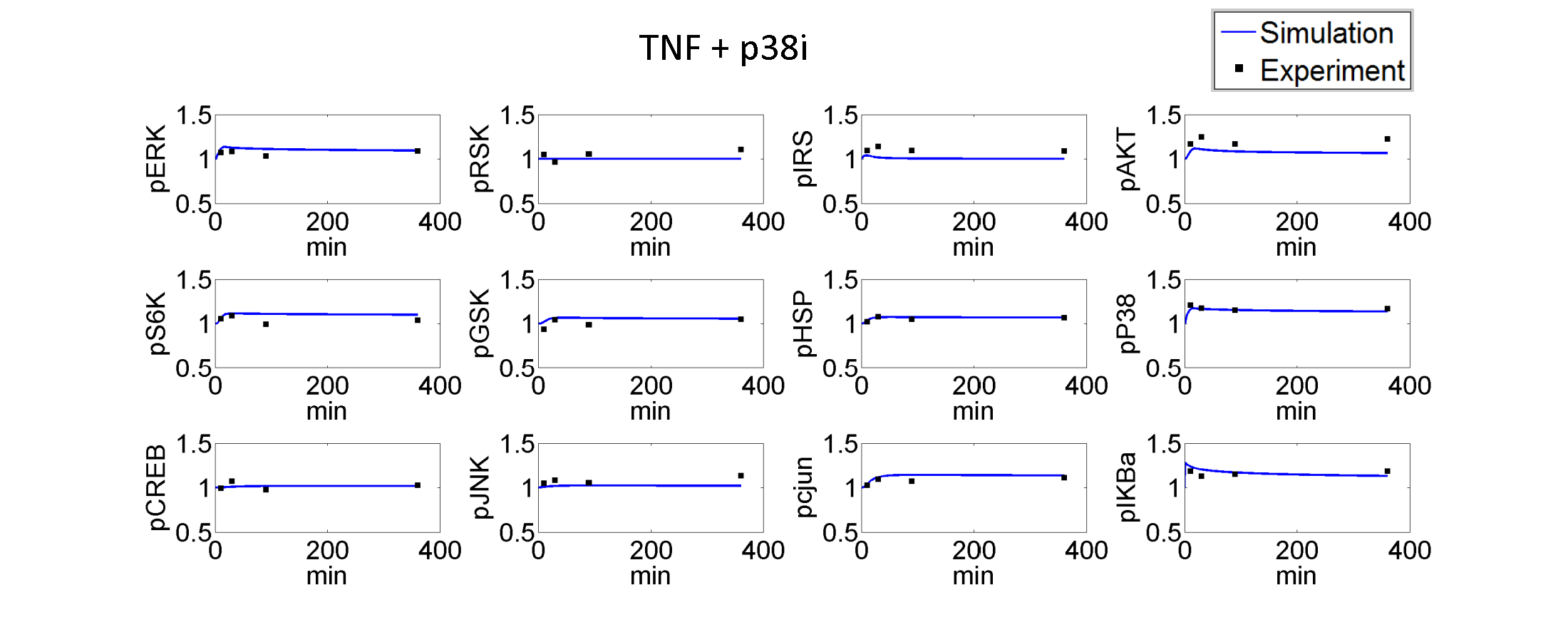

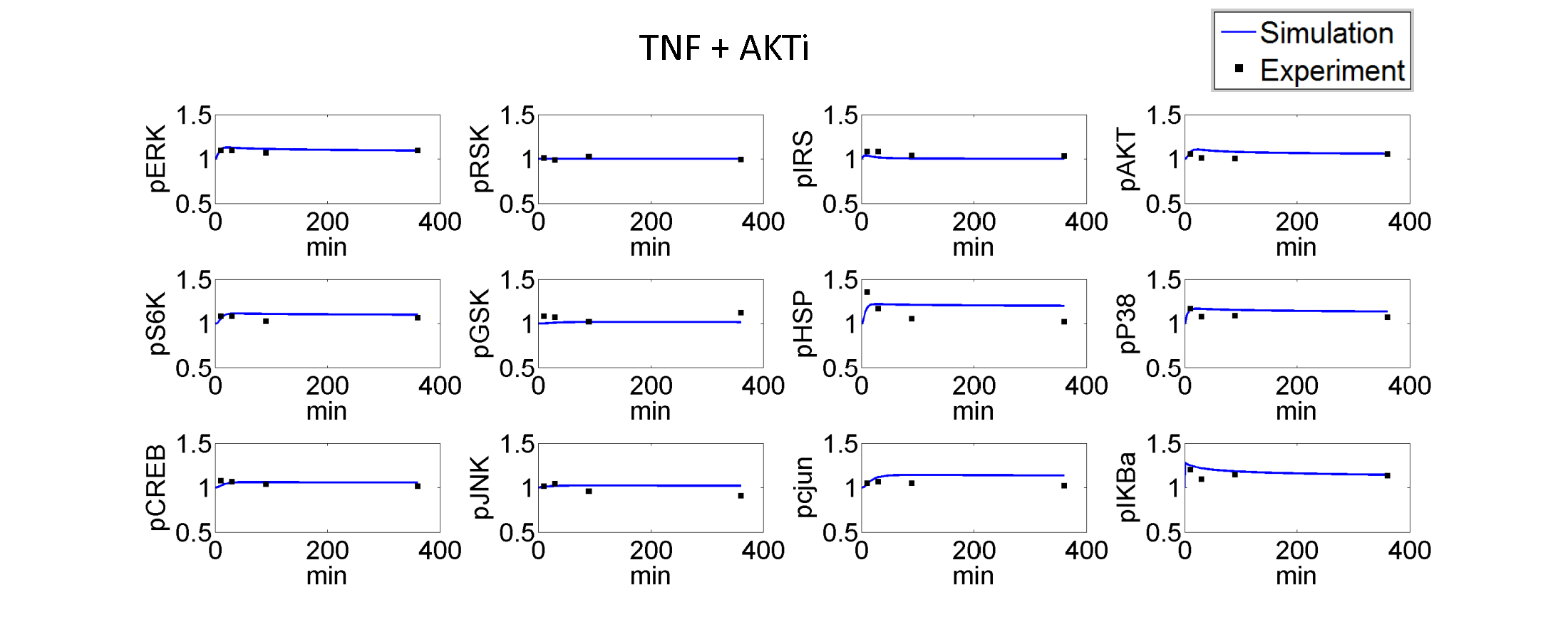


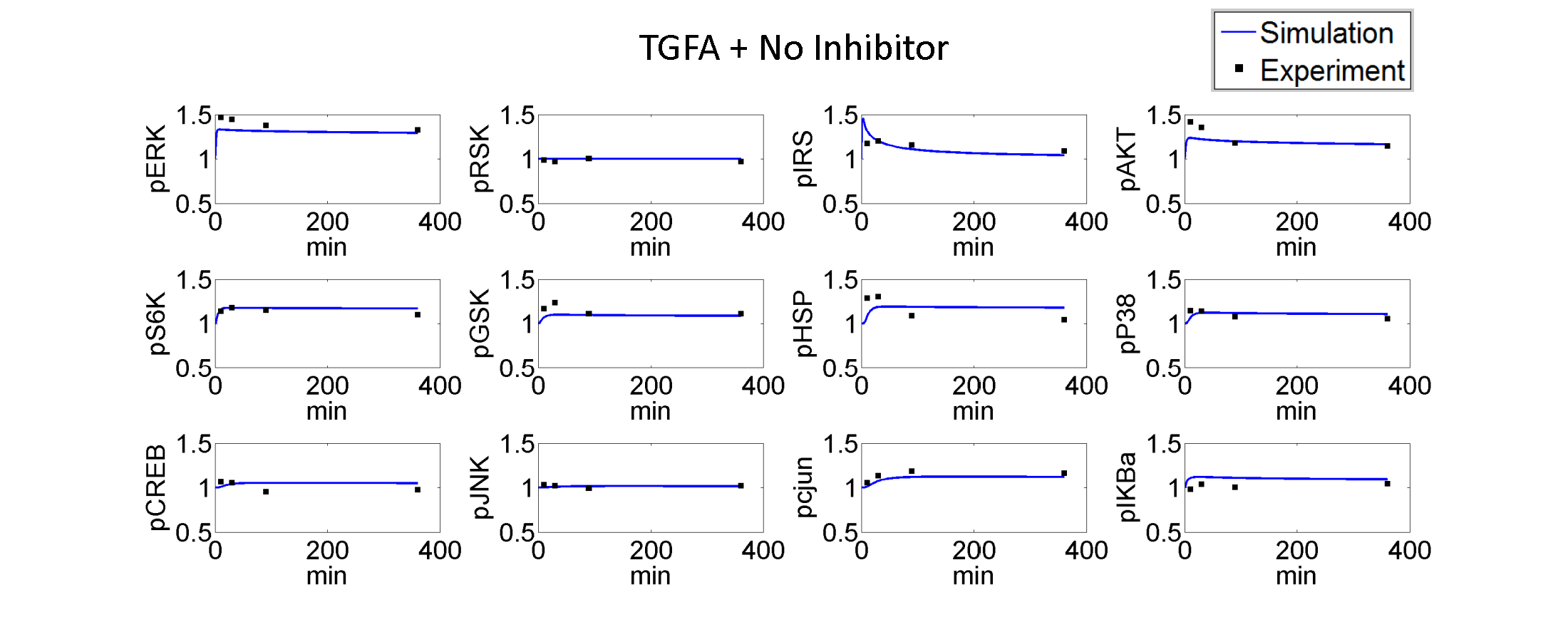

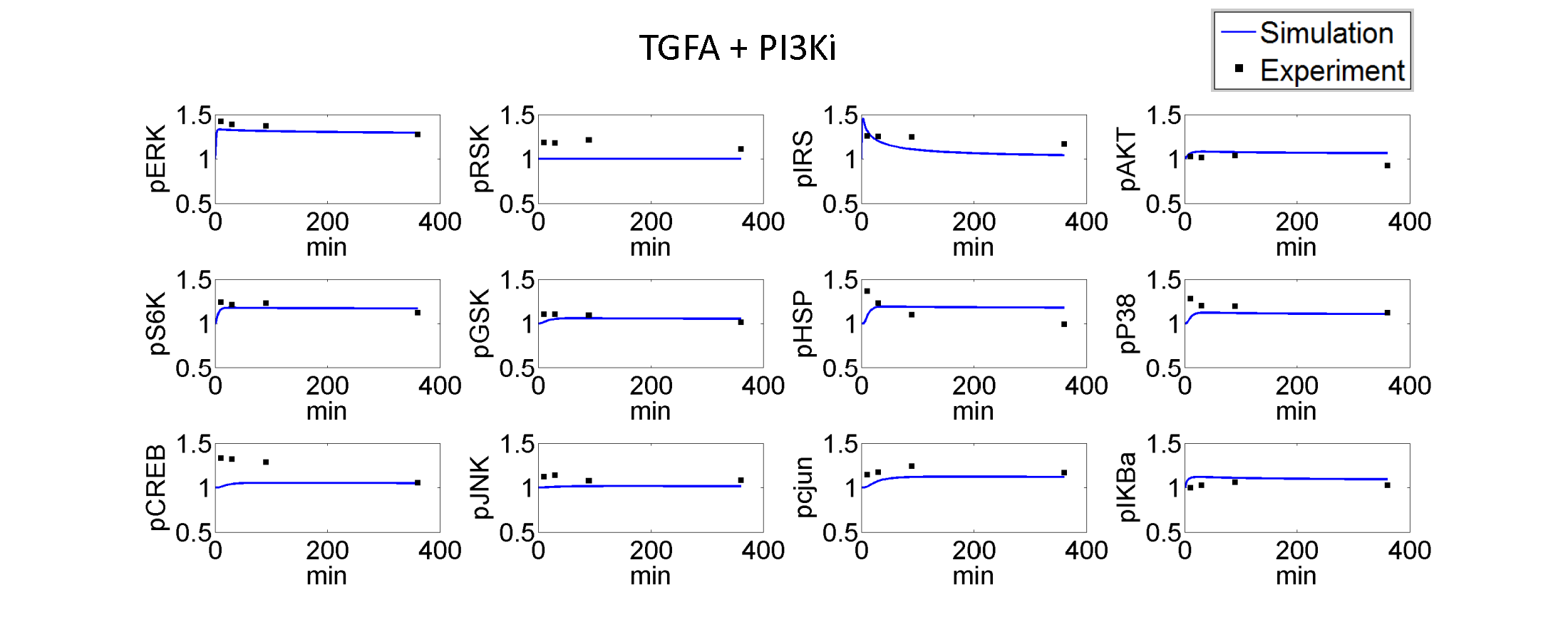

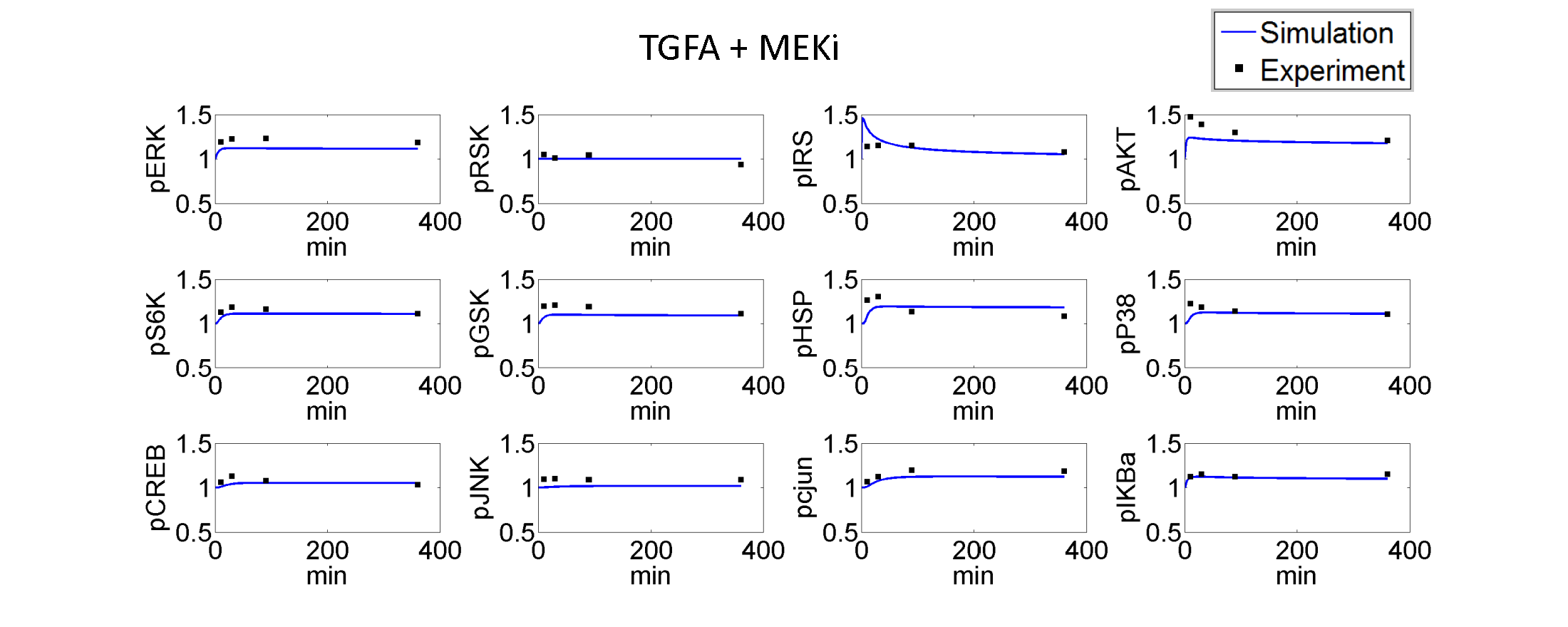


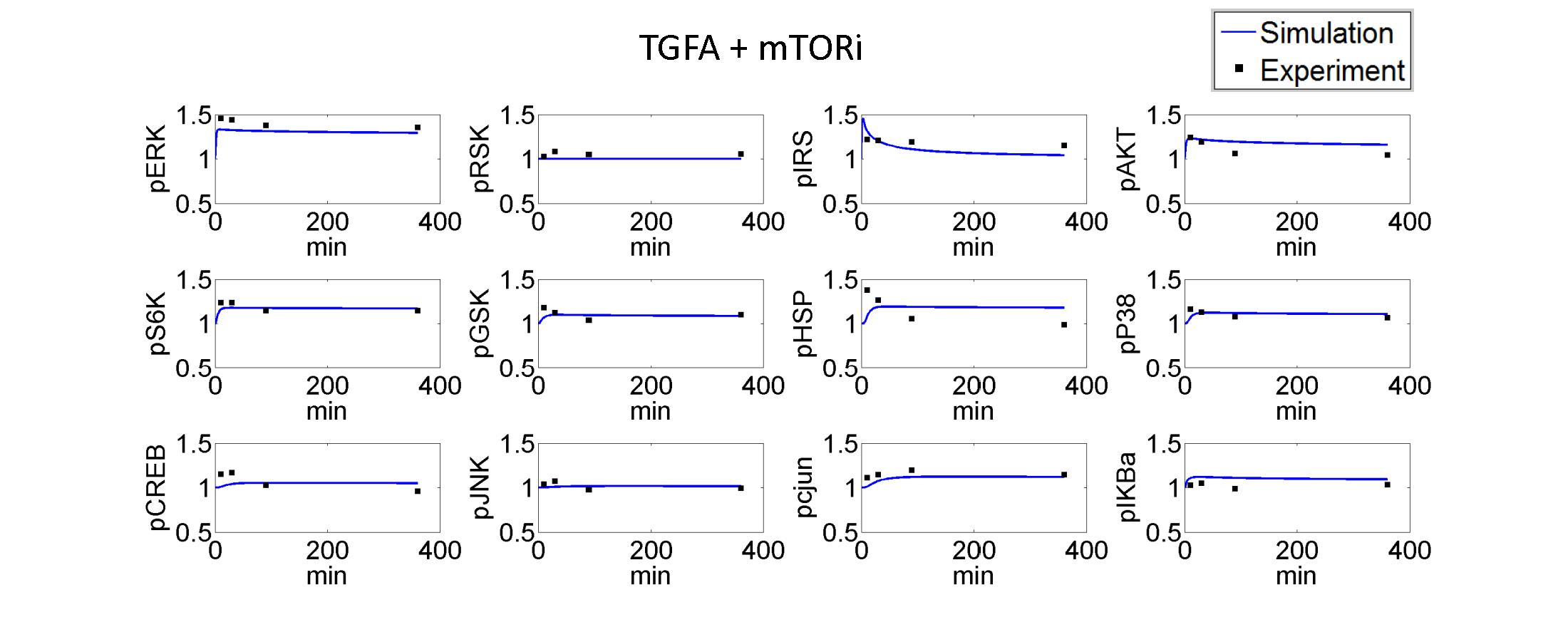

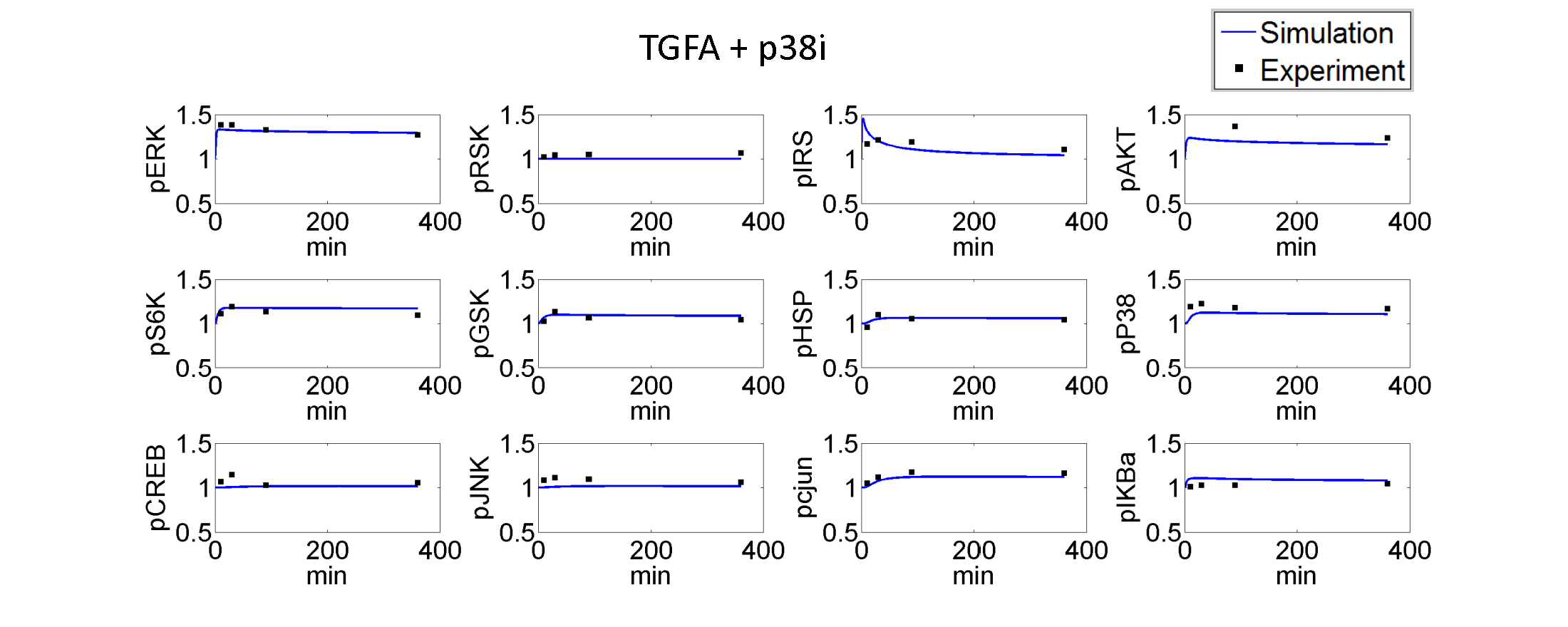

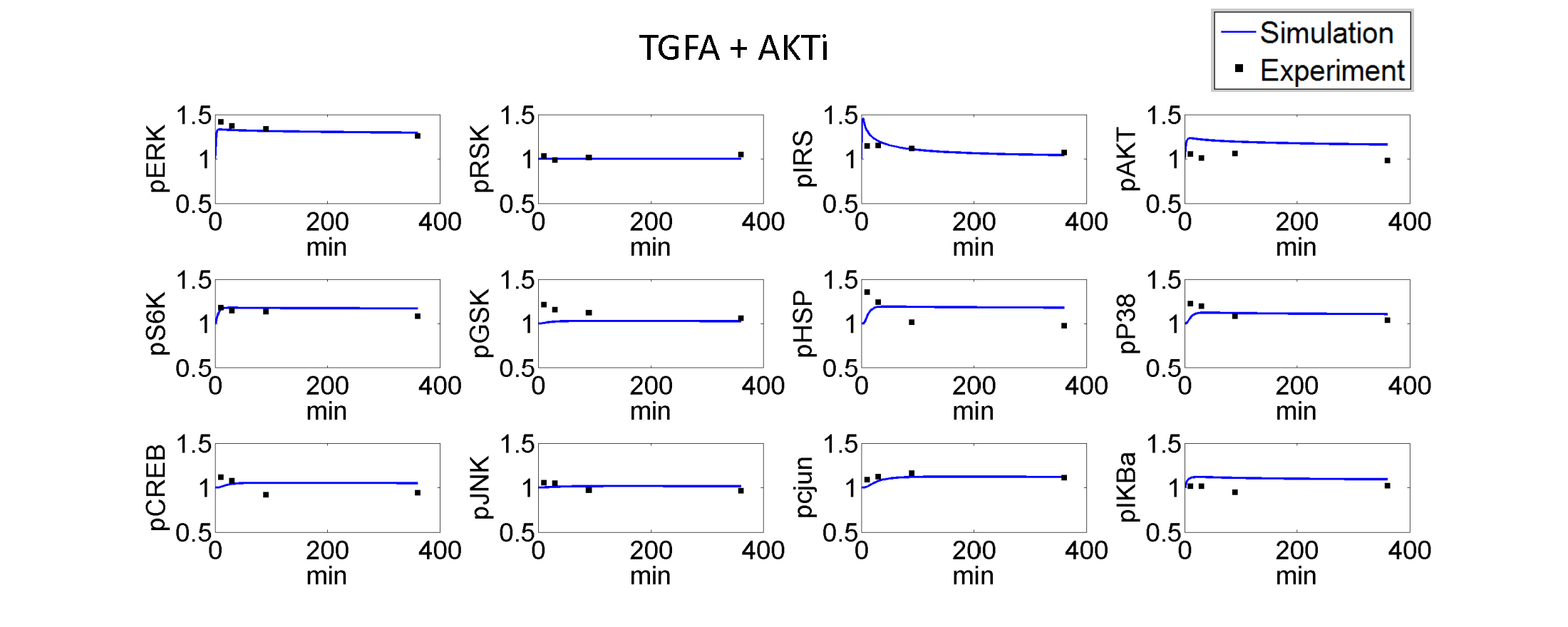


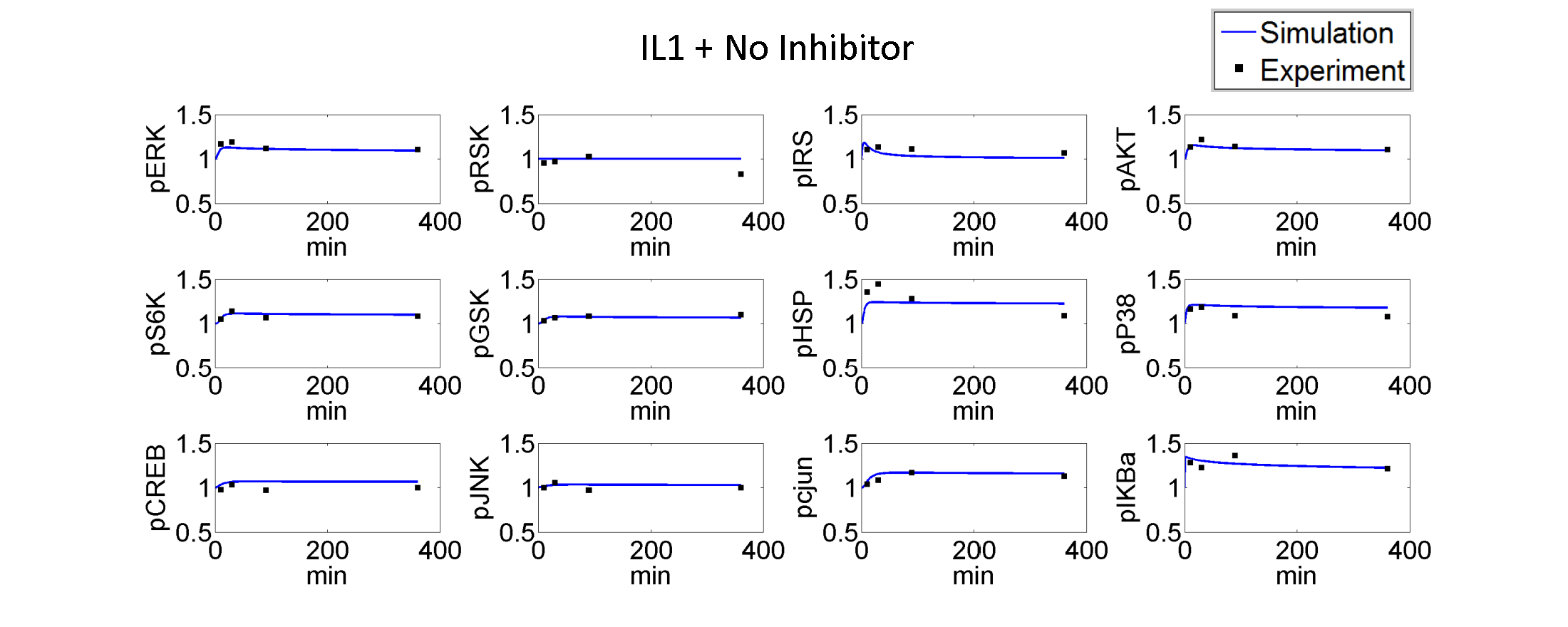

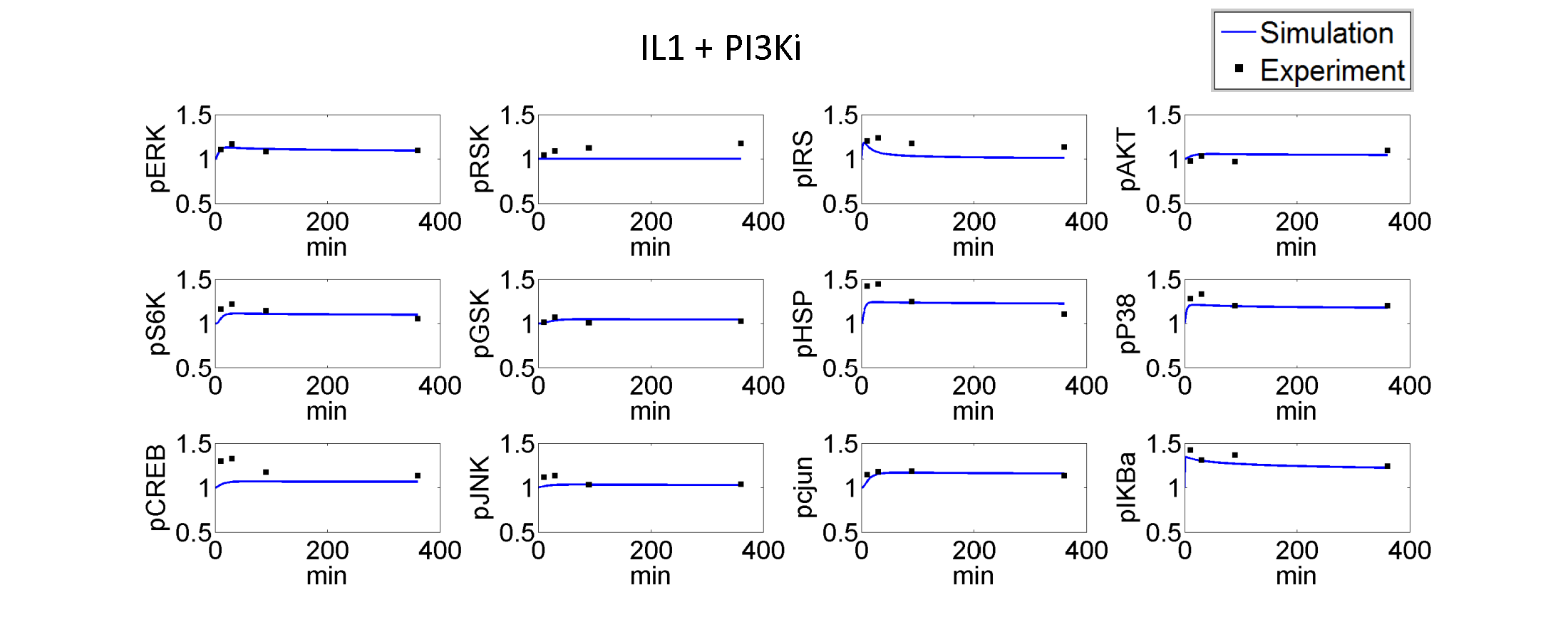

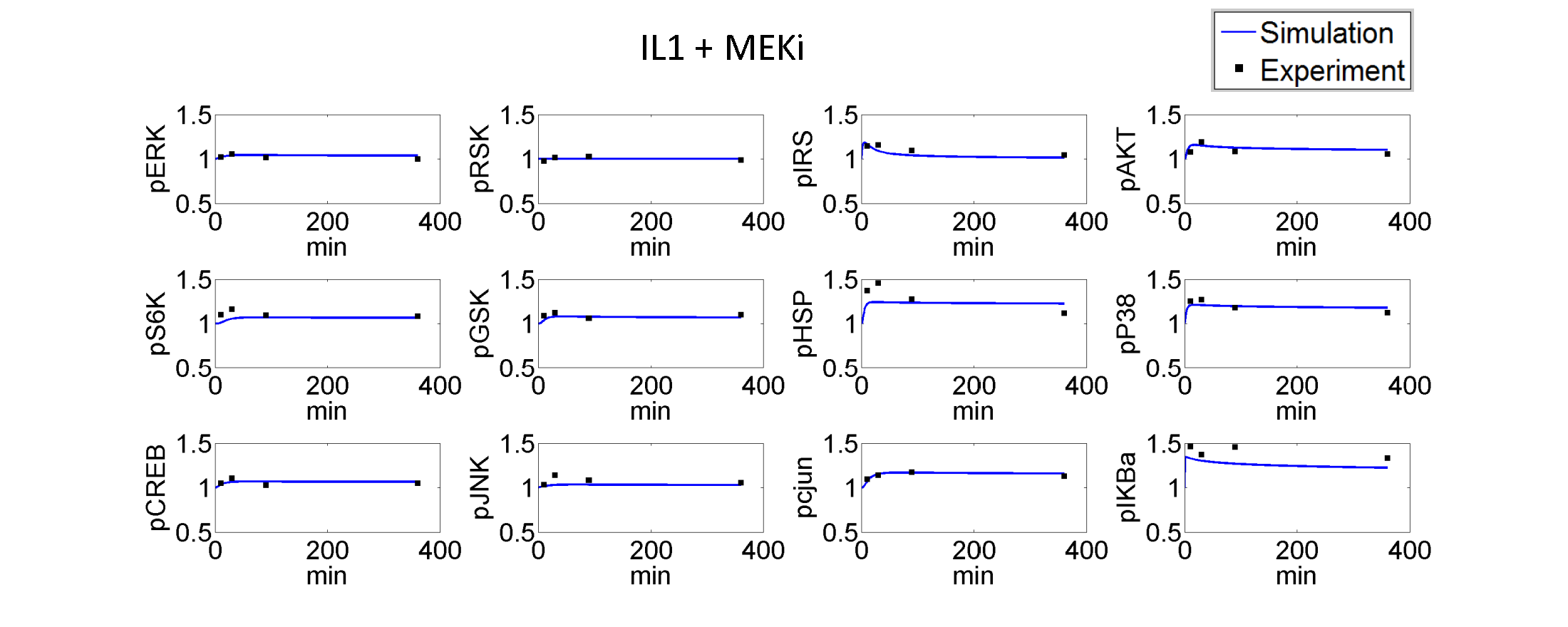


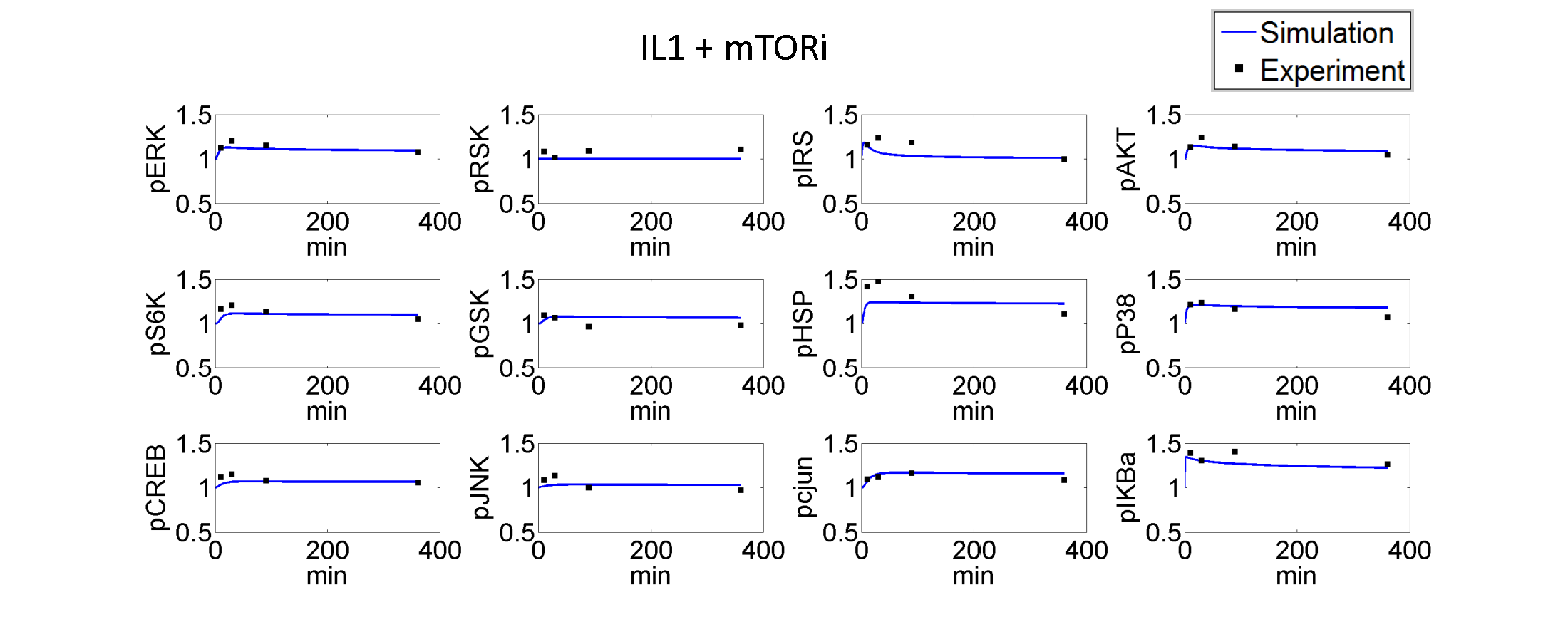

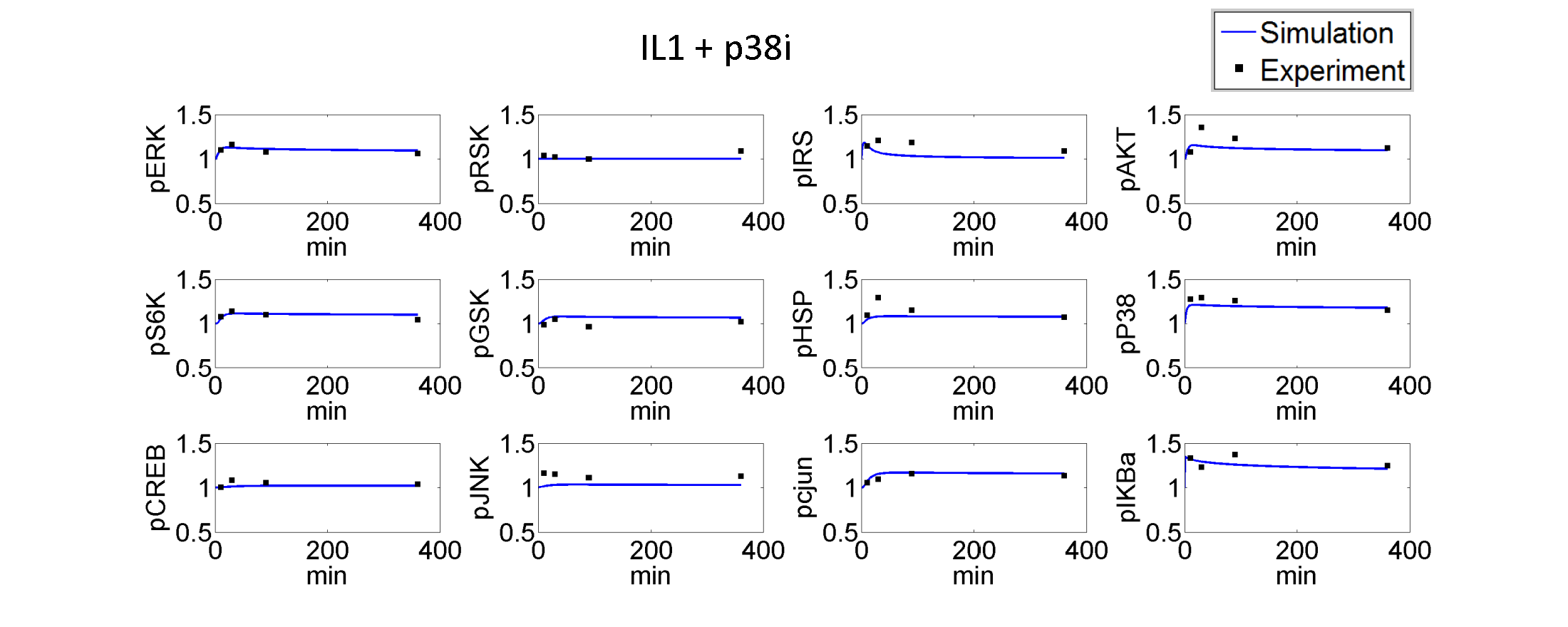

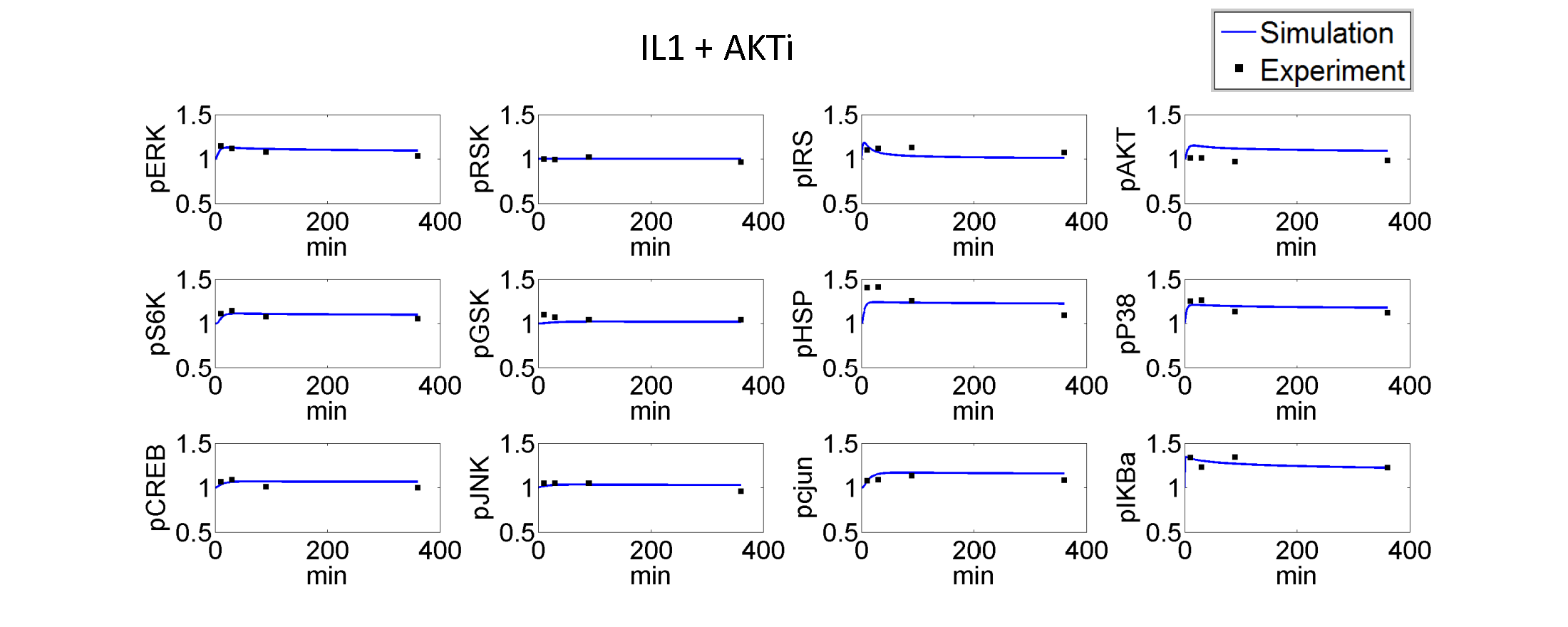


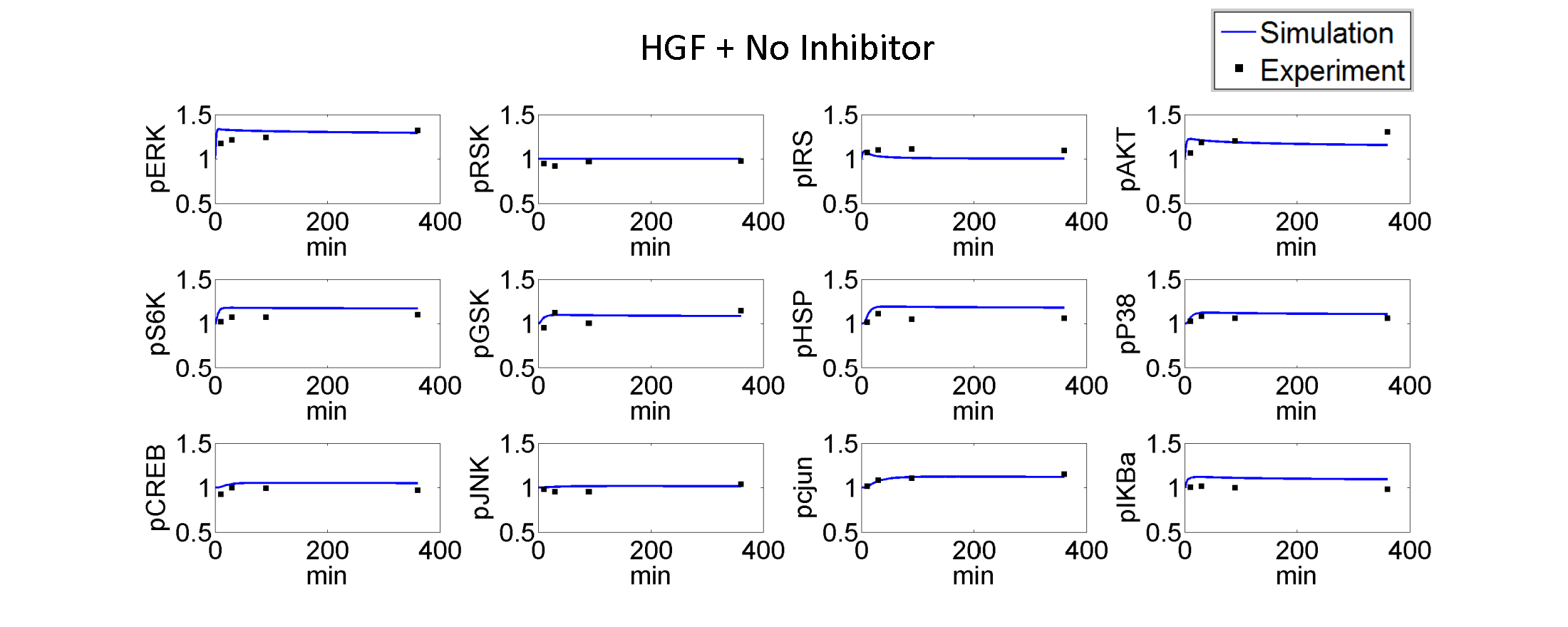

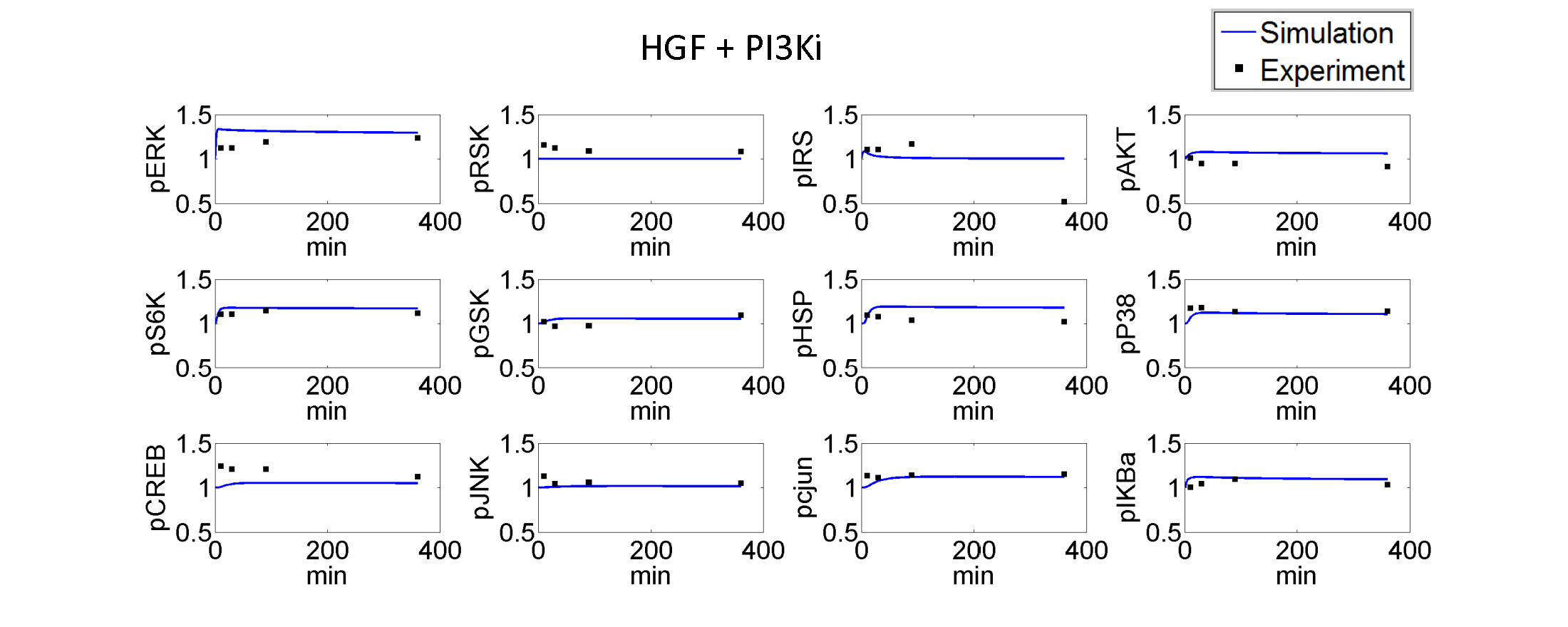

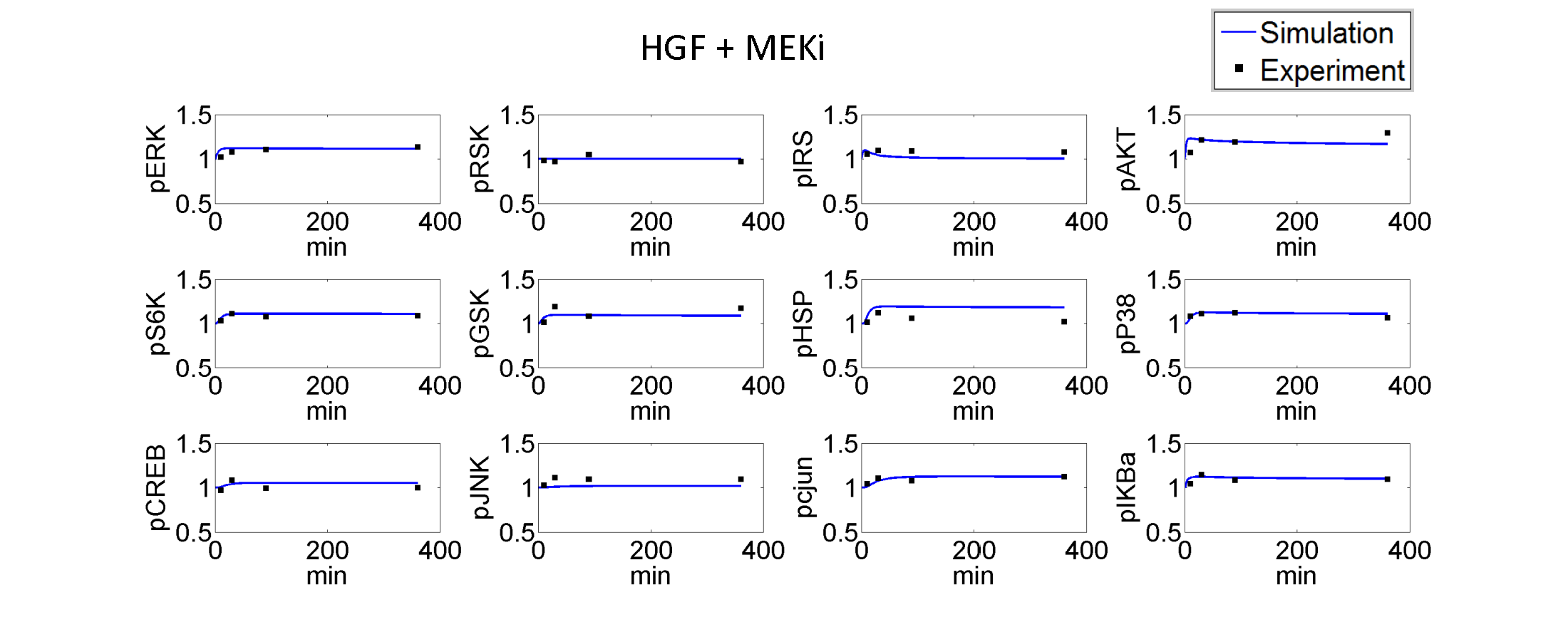


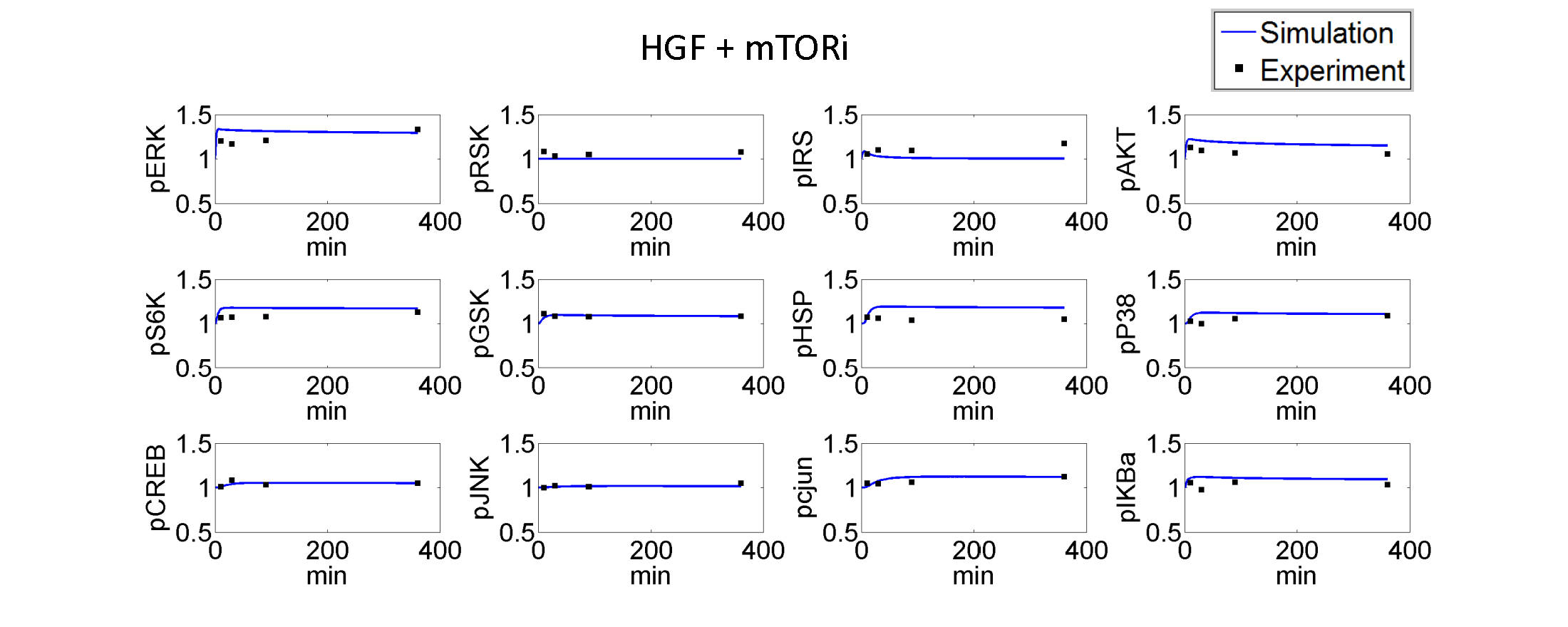

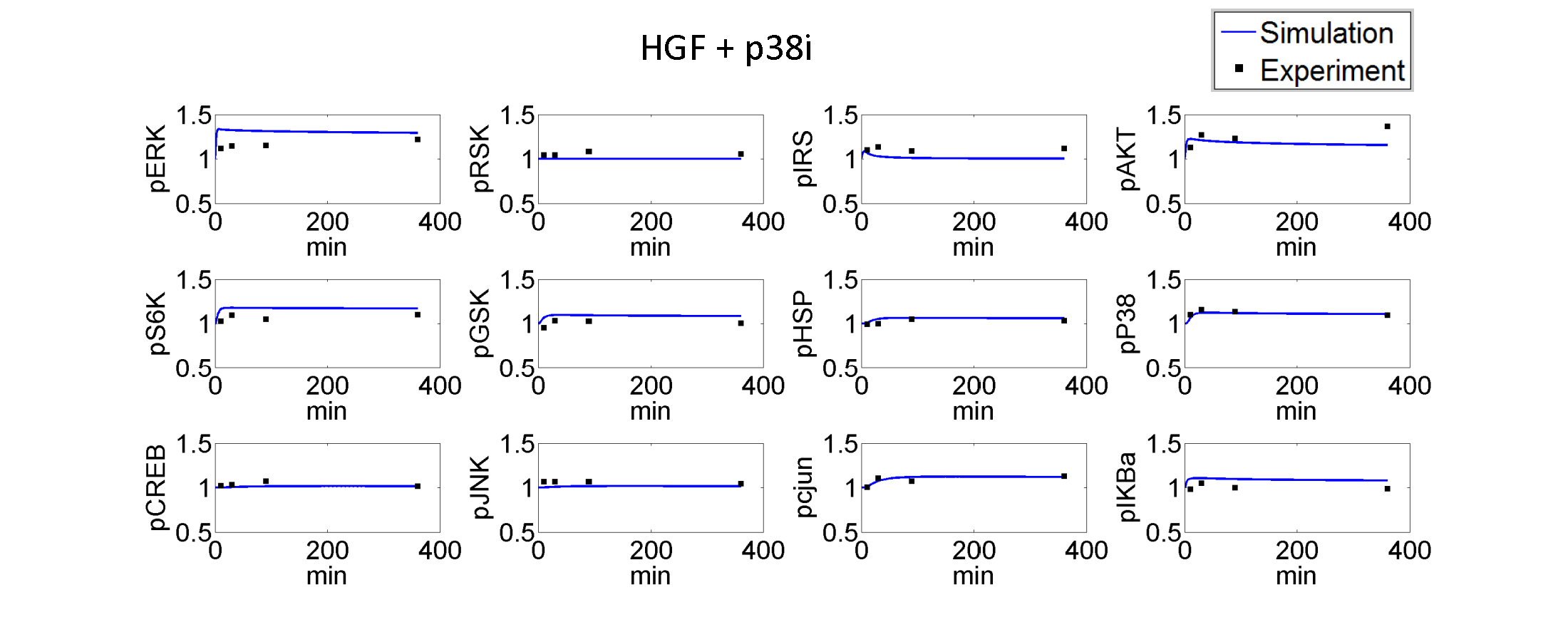

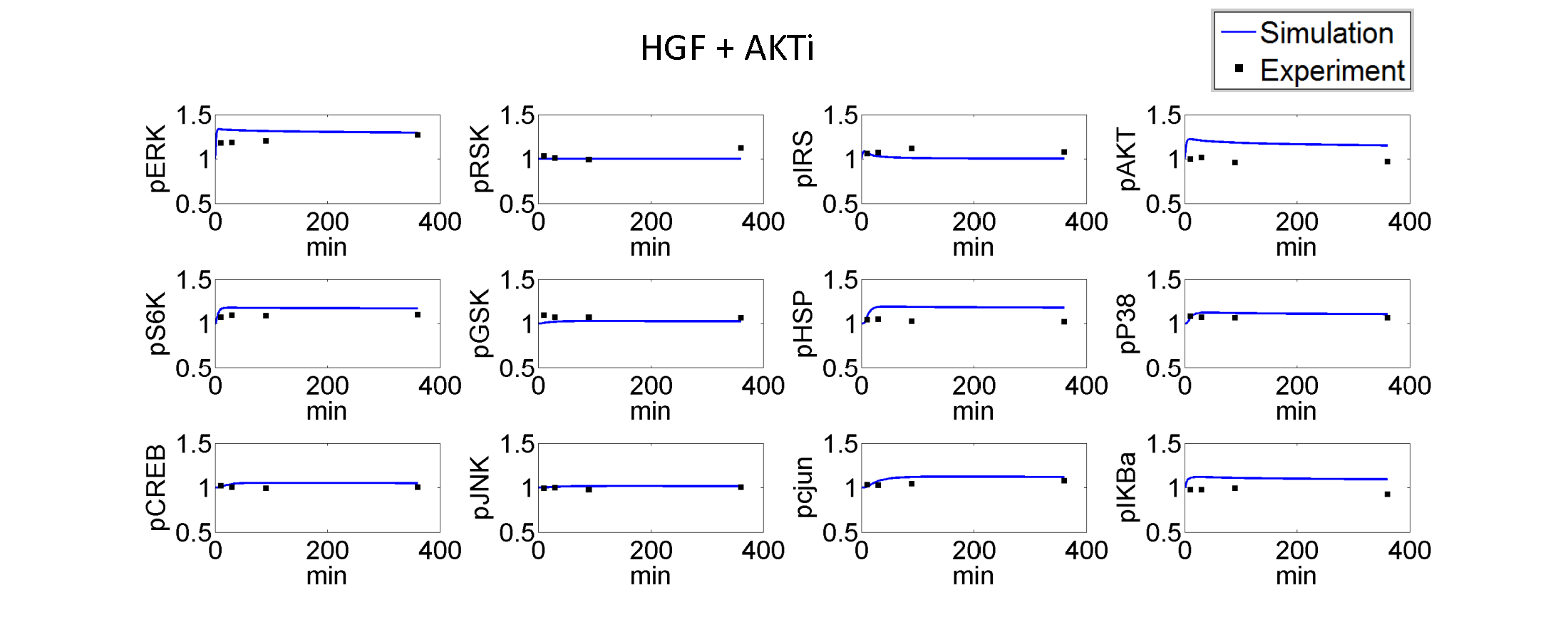


Figure S3 Simulation results from primary human hepatocyte pathway model trained by the cue signal response data.
