# Supplementary material for: Systematically Studying Kinase Inhibitor Induced Signaling Network Signatures by Integrating Both Therapeutic and Side Effects
Source: PLoS One. 2013 Dec 5;8(12):e80832. doi: 10.1371/journal.pone.0080832 (PMC3855094; doi:10.1371/journal.pone.0080832)
Supplement: Text S1 — Mathematical model for cell cycle related pathway. (DOCX) [file pone.0080832.s004.docx]

**Text S1. Mathematical model for cell cycle related pathway**

$\frac{d[PKC]}{dt}=k_{ALK/PKC}\cdot\frac{[ALK]^{n_{ALK/PKC}}}{H_{ALK/PKC}^{n_{ALK/PKC}}+[ALK]^{n_{ALK/PKC}}}+k_{FGFR1/PKC}\cdot\frac{[FGFR1]^{n_{FGFR1/PKC}}}{H_{FGFR1/PKC}^{n_{FGFR1/PKC}}+[FGFR1]^{n_{FGFR1/PKC}}}\text{+}k_{LCK/PKC}\cdot\frac{[LCK]^{n_{LCK/PKC}}}{H_{LCK/PKC}^{n_{LCK/PKC}}+[LCK]^{n_{LCK/PKC}}}-d_{PKC}\cdot[PKC]$ $\frac{d[Src]}{dt}=k_{FGFR1/Src}\cdot\frac{[FGFR1]^{n_{FGFR1/Src}}}{H_{FGFR1/Src}^{n_{FGFR1/Src}}+[FGFR1]^{n_{FGFR1/Src}}}-d_{Src}\cdot[Src]$

$\begin{matrix} \frac{d[Ras]}{dt}=k_{PKC/Ras}\cdot\frac{[PKC]^{n_{PKC/Ras}}}{H_{PKC/Ras}^{n_{PKC/Ras}}+[PKC]^{n_{PKC/Ras}}}+k_{LCK/Ras}\cdot\frac{[LCK]^{n_{LCK/Ras}}}{H_{LCK/Ras}^{n_{LCK/Ras}}+[LCK]^{n_{LCK/Ras}}}+k_{Src/Ras}\cdot\frac{[Src]^{n_{Src/Ras}}}{H_{Src/Ras}^{n_{Src/Ras}}+[Src]^{n_{Src/Ras}}}+k_{EGFR/Ras}\cdot\frac{[EGFR]^{n_{EGFR/Ras}}}{H_{EGFR/Ras}^{n_{EGFR/Ras}}+[EGFR]^{n_{EGFR/Ras}}} \\ +k_{BcrAbl/Ras}\cdot\frac{[BcrAbl]^{n_{BcrAbl/Ras}}}{H_{BcrAbl/Ras}^{n_{BcrAbl/Ras}}+[BcrAbl]^{n_{BcrAbl/Ras}}}+k_{cMet/Ras}\cdot\frac{[cMet]^{n_{cMet/Ras}}}{H_{cMet/Ras}^{n_{cMet/Ras}}+[cMet]^{n_{cMet/Ras}}}-d_{Ras}\cdot[Ras] \end{matrix}$ $\frac{d[JAK]}{dt}=k_{FGFR1/JAK}\cdot\frac{[FGFR1]^{n_{FGFR1/JAK}}}{H_{FGFR1/JAK}^{n_{FGFR1/JAK}}+[FGFR1]^{n_{FGFR1/JAK}}}-d_{JAK}\cdot[JAK]$

$$\frac{d[PI3K]}{dt}=k_{Ras/PI3K}\cdot\frac{[Ras]^{n_{Ras/PI3K}}}{H_{Ras/PI3K}^{n_{Ras/PI3K}}+[Ras]^{n_{Ras/PI3K}}}+k_{LCK/PI3K}\cdot\frac{[LCK]^{n_{LCK/PI3K}}}{H_{LCK/PI3K}^{n_{LCK/PI3K}}+[LCK]^{n_{LCK/PI3K}}}+k_{ITK/PI3K}\cdot\frac{[ITK]^{n_{ITK/PI3K}}}{H_{ITK/PI3K}^{n_{ITK/PI3K}}+[ITK]^{n_{ITK/PI3K}}}-d_{PI3K}\cdot[PI3K]$$

$$\frac{d[STAT3]}{dt}=k_{JAK/STAT3}\cdot\frac{[JAK]^{n_{JAK/STAT3}}}{H_{JAK/STAT3}^{n_{JAK/STAT3}}+[JAK]^{n_{JAK/STAT3}}}+k_{Ras/STAT3}\cdot\frac{[Ras]^{n_{Ras/STAT3}}}{H_{Ras/STAT3}^{n_{Ras/STAT3}}+[Ras]^{n_{Ras/STAT3}}}-d_{STAT3}\cdot[STAT3]$$

$$\frac{d[p38]}{dt}=k_{PKC/p38}\cdot\frac{[PKC]^{n_{PKC/p38}}}{H_{PKC/p38}^{n_{PKC/p38}}+[PKC]^{n_{PKC/p38}}}-d_{p38}\cdot[p38]$$

$$\frac{d[BRAF]}{dt}=k_{PI3K/BRAF}\cdot\frac{[PI3K]^{n_{PI3K/BRAF}}}{H_{PI3K/BRAF}^{n_{PI3K/BRAF}}+[PI3K]^{n_{PI3K/BRAF}}}-d_{BRAF}\cdot[BRAF]$$

$$\frac{d[ERK]}{dt}=k_{BRAF/ERK}\cdot\frac{[BRAF]^{n_{BRAF/ERK}}}{H_{BRAF/ERK}^{n_{BRAF/ERK}}+[BRAF]^{n_{BRAF/ERK}}}-d_{ERK}\cdot[ERK]$$

$$\frac{d[AKT]}{dt}=k_{HSP90/AKT}\cdot\frac{[HSP90]^{n_{HSP90/AKT}}}{H_{HSP90/AKT}^{n_{HSP90/AKT}}+[HSP90]^{n_{HSP90/AKT}}}+k_{PI3K/AKT}\cdot\frac{[PI3K]^{n_{PI3K/AKT}}}{H_{PI3K/AKT}^{n_{PI3K/AKT}}+[PI3K]^{n_{PI3K/AKT}}}-d_{AKT}\cdot[AKT]$$

$$\frac{d[Cdc25C]}{dt}=k_{BRAF/Cdc25C}\cdot\frac{[BRAF]^{n_{BRAF/Cdc25C}}}{H_{BRAF/Cdc25C}^{n_{BRAF/Cdc25C}}+[BRAF]^{n_{BRAF/Cdc25C}}}+k_{PLK/Cdc25C}\cdot\frac{[PLK]^{n_{PLK/Cdc25C}}}{H_{PLK/Cdc25C}^{n_{PLK/Cdc25C}}+[PLK]^{n_{PLK/Cdc25C}}}-d_{Cdc25C}\cdot[Cdc25C]$$

$$\frac{d[Wee1]}{dt}=k_{AKT/Wee1}\cdot\frac{[AKT]^{-n_{AKT/Wee1}}}{H_{AKT/Wee1}^{-n_{AKT/Wee1}}+[AKT]^{-n_{AKT/Wee1}}}-d_{Wee1}\cdot[Wee1]$$

$$\frac{d[PLK]}{dt}=k_{Aurora/PLK}\cdot\frac{[Aurora]^{n_{Aurora/PLK}}}{H_{Aurora/PLK}^{n_{Aurora/PLK}}+[Aurora]^{n_{Aurora/PLK}}}+k_{CycB/PLK}\cdot\frac{[CycB]^{n_{CycB/PLK}}}{H_{CycB/PLK}^{n_{CycB/PLK}}+[CycB]^{n_{CycB/PLK}}}-d_{PLK}\cdot[PLK]$$

$\begin{matrix} \frac{d[CycD]}{dt}=k_{STAT3/CycD}\cdot\frac{[STAT3]^{n_{STAT3/CycD}}}{H_{STAT3/CycD}^{n_{STAT3/CycD}}+[STAT3]^{n_{STAT3/CycD}}}+k_{p38/CycD}\cdot\frac{[p38]^{-n_{p38/CycD}}}{H_{p38/CycD}^{-n_{p38/CycD}}+[p38]^{-n_{p38/CycD}}}+k_{ERK/CycD}\cdot\frac{[ERK]^{n_{ERK/CycD}}}{H_{ERK/CycD}^{n_{ERK/CycD}}+[ERK]^{n_{ERK/CycD}}} \\ -d_{CycD}\cdot[CycD] \end{matrix}$ $\frac{d[CycE]}{dt}=k_{AKT/CycE}\cdot\frac{[AKT]^{n_{AKT/CycE}}}{H_{AKT/CycE}^{n_{AKT/CycE}}+[AKT]^{n_{AKT/CycE}}}+k_{p38/CycE}\cdot\frac{[p38]^{-n_{p38/CycE}}}{H_{p38/CycE}^{-n_{p38/CycE}}+[p38]^{-n_{p38/CycE}}}-d_{CycE}\cdot[CycE]$

$$\frac{d[CycA]}{dt}=k_{STAT3/CycA}\cdot\frac{[STAT3]^{n_{STAT3/CycA}}}{H_{STAT3/CycA}^{n_{STAT3/CycA}}+[STAT3]^{n_{STAT3/CycA}}}-d_{CycA}\cdot[CycA]$$

$\begin{matrix} \frac{d[CycB]}{dt}=k_{ERK/CycB}\cdot\frac{[ERK]^{n_{ERK/CycB}}}{H_{ERK/CycB}^{n_{ERK/CycB}}+[ERK]^{n_{ERK/CycB}}}+k_{Wee1/CycB}\cdot\frac{[Wee1]^{-n_{Wee1/CycB}}}{H_{Wee1/CycB}^{-n_{Wee1/CycB}}+[Wee1]^{-n_{Wee1/CycB}}}+k_{Cdc25C/CycB}\cdot\frac{[Cdc25C]^{n_{Cdc25C/CycB}}}{H_{Cdc25C/CycB}^{n_{Cdc25C/CycB}}+[Cdc25C]^{n_{Cdc25C/CycB}}} \\ -d_{CycB}\cdot[CycB] \end{matrix}$ $\frac{d[G1]}{dt}=k_{CycD/G1}\cdot\frac{[CycD]^{n_{CycD/G1}}}{H_{CycD/G1}^{n_{CycD/G1}}+[CycD]^{n_{CycD/G1}}}\cdot M-k_{CycE/S}\cdot\frac{[CycE]^{n_{CycE/S}}}{H_{CycE/S}^{n_{CycE/S}}+[CycE]^{n_{CycE/S}}}\cdot G1-d_{G1}\cdot[G1]$

$$\frac{d[S]}{dt}=k_{CycE/S}\cdot\frac{[CycE]^{n_{CycE/S}}}{H_{CycE/S}^{n_{CycE/S}}+[CycE]^{n_{CycE/S}}}\cdot G1-k_{CycA/G2}\cdot\frac{[CycA]^{n_{CycA/G2}}}{H_{CycA/G2}^{n_{CycA/G2}}+[CycA]^{n_{CycA/G2}}}\cdot S-d_{S}\cdot[S]$$

$$\frac{d[G2]}{dt}=k_{CycA/G2}\cdot\frac{[CycA]^{n_{CycA/G2}}}{H_{CycA/G2}^{n_{CycA/G2}}+[CycA]^{n_{CycA/G2}}}\cdot S-k_{CycB/M}\cdot\frac{[CycB]^{n_{CycB/M}}}{H_{CycB/M}^{n_{CycB/M}}+[CycB]^{n_{CycB/M}}}\cdot G2-d_{G2}\cdot[G2]$$

$$\frac{d[M]}{dt}=k_{CycB/M}\cdot\frac{[CycB]^{n_{CycB/M}}}{H_{CycB/M}^{n_{CycB/M}}+[CycB]^{n_{CycB/M}}}\cdot G2-k_{CycD/G1}\cdot\frac{[CycD]^{n_{CycD/G1}}}{H_{CycD/G1}^{n_{CycD/G1}}+[CycD]^{n_{CycD/G1}}}\cdot M-d_{M}\cdot[M]$$
