# Supplementary material for: Systematically Studying Kinase Inhibitor Induced Signaling Network Signatures by Integrating Both Therapeutic and Side Effects
Source: PLoS One. 2013 Dec 5;8(12):e80832. doi: 10.1371/journal.pone.0080832 (PMC3855094; doi:10.1371/journal.pone.0080832)
Supplement: Text S2 — Mathematical model for primary human hepatocyte pathway. (DOCX) [file pone.0080832.s005.docx]

**Text S2. Mathematical model for primary human hepatocyte pathway**

$\begin{matrix} \frac{d[pRas]}{dt}=k_{TGFa/Ras}\cdot[TGFa]\cdot([tRas]-[pRas])+k_{INS/Ras}\cdot[INS]\cdot([tRas]-[pRas])+k_{IGF/Ras}\cdot[IGF]\cdot([tRas]-[pRas]) \\ +k_{HGF/Ras}\cdot[HGF]\cdot([tRas]-[pRas])-d_{pRas}\cdot[pRas] \end{matrix}$ $\frac{d[pRaf]}{dt}=k_{Ras/Raf}\cdot[pRas]\cdot([tRaf]-[pRaf])-k_{ERK/Raf}\cdot[pERK]\cdot[pRaf]-k_{AKT/Raf}\cdot[pAKT]\cdot[pRaf]-d_{pRaf}\cdot[pRaf]$

$$\frac{d[pMEK]}{dt}=k_{Raf/MEK}\cdot[pRaf]\cdot([tMEK]-[pMEK])-k_{ERK/MEK}\cdot[pERK][pMEK]-d_{pMEK}\cdot[pMEK]$$

$$\frac{d[pERK]}{dt}=k_{MEK/ERK}\cdot[pMEK]\cdot([tERK]-[pERK])-d_{pERK}\cdot[pERK]$$

$$\frac{d[pp90RSK]}{dt}=k_{ERK/p90RSK}\cdot[pERK]\cdot([tp90RSK]-[pp90RSK])-d_{pp90RSK}\cdot[pp90RSK]$$

$\begin{matrix} \frac{d[pPI3K]}{dt}=k_{TGFa/PI3K}\cdot[TGFa]\cdot([tPI3K]-[pPI3K])+k_{Ras/PI3K}\cdot[pRas]\cdot([tPI3K]-[pPI3K])+k_{HGF/PI3K}\cdot[HGF]\cdot([tPI3K]-[pPI3K]) \\ +k_{IRS/PI3K}\cdot[IRS]\cdot([tPI3K]-[pPI3K])-d_{pPI3K}\cdot[pPI3K] \end{matrix}$ $\frac{d[pAKT]}{dt}=k_{PI3K/AKT}\cdot[pPI3K]\cdot([tAKT]-[pAKT])+k_{HSP27/AKT}\cdot[pHSP27]\cdot([tAKT]-[pAKT])-d_{pAKT}\cdot[pAKT]$

$$\frac{d[mTORC]}{dt}=k_{AKT/mTORC}\cdot[pAKT]\cdot([tmTOR]-[mTORC])-k_{MEK/mTORC}\cdot[pMEK]\cdot[mTORC]-d_{mTORC}\cdot[mTORC]$$

$\begin{matrix} \frac{d[pp70S6K]}{dt}=k_{ERK/p70S6K}\cdot[pERK]\cdot([tp70S6K]-[pp70S6K])+k_{mTORC/p70S6K}\cdot[mTORC]\cdot([tp70S6K]-[pp70S6K]) \\ -d_{pp70S6K}\cdot[pp70S6K] \end{matrix}$ $\frac{d[pIRS]}{dt}=k_{INS/IRS}\cdot[INS]\cdot([tIRS]-[pIRS])+k_{IGF/IRS}\cdot[IGF]\cdot([tIRS]-[pIRS])-k_{JNK/IRS}\cdot[pJNK]\cdot[pIRS]-d_{pIRS}\cdot[pIRS]$

$$\frac{d[pHSP27]}{dt}=k_{p38/HSP27}\cdot[pp38]\cdot([tHSP27]-[pHSP27])-d_{pHSP27}\cdot[pHSP27]$$

$\begin{matrix} \frac{d[pMEKK]}{dt}=k_{Ras/MEKK}\cdot[pRas]\cdot([tMEKK]-[pMEKK])+k_{IL1/MEKK}\cdot[IL1]\cdot([tMEKK]-[pMEKK]) \\ +k_{TNFa/MEKK}\cdot[TNFa]\cdot([tMEKK]-[pMEKK])-d_{pMEKK}\cdot[pMEKK] \end{matrix}$ $\frac{d[pMKK3]}{dt}=k_{MEKK/MKK3}\cdot[pMEKK]\cdot([tMKK3]-[pMKK3])+k_{TAK/MKK3}\cdot[pTAK]\cdot([tMKK3]-[pMKK3])-d_{pMKK3}\cdot[pMKK3]$

$$\frac{d[pp38]}{dt}=k_{MKK3/p38}\cdot[pMKK3]\cdot([tp38]-[pp38])-d_{pp38}\cdot[pp38]$$

$$\frac{d[pCREB]}{dt}=k_{p90RSK/CREB}\cdot[pp90RSK]\cdot([tCREB]-[pCREB])+k_{p38/CREB}\cdot[pp38]\cdot([tCREB]-[pCREB])-d_{pCREB}\cdot[pCREB]$$

$$\frac{d[pTAK]}{dt}=k_{IL1/TAK}\cdot[IL1]\cdot([tTAK]-[pTAK])-d_{pTAK}\cdot[pTAK]$$

$$\frac{d[pMKK4]}{dt}=k_{MEKK/MKK4}\cdot[pMEKK]\cdot([tMKK4]-[pMKK4])+k_{TAK/MKK4}\cdot[pTAK]\cdot([tMKK4]-[pMKK4])-d_{pMKK4}\cdot[pMKK4]$$

$$\frac{d[pJNK]}{dt}=k_{MKK4/JNK}\cdot[pMKK4]\cdot([tJNK]-[pJNK])-d_{pJNK}\cdot[pJNK]$$

$$\frac{d[pCJUN]}{dt}=k_{JNK/CJUN}\cdot[pJNK]\cdot([tCJUN]-[pCJUN])-d_{pCJUN}\cdot[pCJUN]$$

$\begin{matrix} \frac{d[pIKK]}{dt}=k_{MEKK/IKK}\cdot[pMEKK]\cdot([tIKK]-[pIKK])+k_{TAK/IKK}\cdot[pTAK]\cdot([tIKK]-[pIKK])+k_{TNFa/IKK}\cdot[TNFa]\cdot([tIKK]-[pIKK]) \\ -d_{pIKK}\cdot[pIKK] \end{matrix}$ $\frac{d[NFkBC]}{dt}=k_{IKB/NFkB}\cdot[IKB]\cdot[NFkB]-k_{IKK/NFkBC}\cdot[pIKK]\cdot[NFkBC]$

$$\frac{d[IKB]}{dt}=k_{NFkB/IKB}\cdot[NFkB]+k_{IKK/NFkBC}\cdot[pIKK]\cdot[NFkBC]-k_{IKB/NFkB}\cdot[IKB]\cdot[NFkB]-d_{IKB}\cdot[IKB]$$

$$\frac{d[NFkB]}{dt}=k_{IKK/NFkBC}\cdot[pIKK]\cdot[NFkBC]-k_{IKB/NFkB}\cdot[IKB]\cdot[NFkB]$$
